# Network-based analysis of oligodendrogliomas predicts novel cancer gene candidates within the region of the 1p/19q co-deletion

**DOI:** 10.1186/s40478-018-0544-y

**Published:** 2018-06-11

**Authors:** Josef Gladitz, Barbara Klink, Michael Seifert

**Affiliations:** 1 Institute for Medical Informatics and Biometry, Carl Gustav Carus Faculty of Medicine, Technische Universität Dresden, Dresden, Germany; 2 Institute for Clinical Genetics, Carl Gustav Carus Faculty of Medicine, Technische Universität Dresden, Dresden, Germany; 3 National Center for Tumor Diseases, Dresden, Germany

**Keywords:** Oligodendrogliomas, 1p/19q co-deletion, Network biology, Network inference, Network propagation, Cancer genomics, Bioinformatics, Computational systems biology

## Abstract

Oligodendrogliomas are primary human brain tumors with a characteristic 1p/19q co-deletion of important prognostic relevance, but little is known about the pathology of this chromosomal mutation. We developed a network-based approach to identify novel cancer gene candidates in the region of the 1p/19q co-deletion. Gene regulatory networks were learned from gene expression and copy number data of 178 oligodendrogliomas and further used to quantify putative impacts of differentially expressed genes of the 1p/19q region on cancer-relevant pathways. We predicted 8 genes with strong impact on signaling pathways and 14 genes with strong impact on metabolic pathways widespread across the region of the 1p/19 co-deletion. Many of these candidates (e.g. *ELTD1*, *SDHB*, *SEPW1*, *SLC17A7*, *SZRD1*, *THAP3*, *ZBTB17*) are likely to push, whereas others (e.g. *CAP1*, *HBXIP*, *KLK6*, *PARK7*, *PTAFR*) might counteract oligodendroglioma development. For example, *ELTD1*, a functionally validated glioblastoma oncogene located on 1p, was overexpressed. Further, the known glioblastoma tumor suppressor *SLC17A7* located on 19q was underexpressed. Moreover, known epigenetic alterations triggered by mutated *SDHB* in paragangliomas suggest that underexpressed *SDHB* in oligodendrogliomas may support and possibly enhance the epigenetic reprogramming induced by the *IDH*-mutation. We further analyzed rarely observed deletions and duplications of chromosomal arms within oligodendroglioma subcohorts identifying putative oncogenes and tumor suppressors that possibly influence the development of oligodendroglioma subgroups. Our in-depth computational study contributes to a better understanding of the pathology of the 1p/19q co-deletion and other chromosomal arm mutations. This might open opportunities for functional validations and new therapeutic strategies.

**Electronic supplementary material:**

The online version of this article (10.1186/s40478-018-0544-y) contains supplementary material, which is available to authorized users.

## Introduction

Between 4 and 8 percent of all primary human brain tumors are classified as oligodendrogliomas [80]. Oligodendrogliomas belong to the class of diffuse gliomas that typically show infiltrative growth into the surrounding brain tissue, relapse, and progression to more aggressive tumors [54]. Histological similarities to normal oligodendrocytes were used for many years to diagnose oligodendrogliomas [47], but pure histological classifications can vary considerably between neuropathologists [14, 76]. Therefore, molecular markers for a more robust classification of oligodendrogliomas have been explored. First, it has been revealed that the majority of oligodendrogliomas showed a recurrent loss of heterozygosity of the chromosomal arms 1p and 19q (1p/19q co-deletion) associated with improved chemotherapy response and longer relapse-free survival [9, 28, 60]. Further, the 1p/19q-co-deletion is always accompanied by heterozygous somatic point mutations of the isocitrate dehydrogenase gene (*IDH1/2*) [37]. These *IDH*-mutations are known to induce the glioma-CpG island methylator phenotype (G-CIMP) [53, 75]. Both characteristic molecular markers (1p/19q co-deletion and *IDH* mutation) have recently been included into the new 2016 World Health Organization (WHO) classification system for tumors of the central nervous system [48]. This new classification utilizes histological features in combination with the co-occurrence of the 1p/19q co-deletion and the *IDH*-mutation to diagnose oligodendrogliomas.

So far, the clinical relevance of the oligodendroglioma-specific 1p/19q co-deletion has been well-studied [9, 10, 28, 33, 69, 78], but the pathogenesis of the recurrent 1p/19q co-deletion still remains elusive. The 1p/19q co-deletion is likely to emerge from an unbalanced translocation between the 1q and 19p arm [29]. This suggests that driver genes could be located in close proximity to the fusion points, but no oncogenic fusion genes have been reported. On the other hand, the recurrent 1p/19q co-deletion suggests that tumor suppressors could be located on the 1p and 19q arm. According to the classical two hit hypothesis, both alleles of a tumor suppressor must be mutated to contribute to oncogenesis [36]. The search for inactivating point mutations on the remaining copies of the 1p and 19q arm identified *FUBP1* located on 1p and *CIC* located on 19q as potential tumor suppressors [8, 20]. But *FUBP1* mutations are only observed in about 29% and *CIC* mutations in about 62% of oligodendrogliomas [69]. This implies that these mutations occur later during tumor development and are therefore not responsible for the initial development of oligodendrogliomas. Moreover, it is likely that haploinsufficiency [16, 63] induced by the 1p/19q co-deletion may contribute to the development of oligodendrogliomas. The loss of one allele of each gene on 1p and 19q could directly contribute to oncogenesis by reduced expression levels or indirectly by alterations of regulatory networks. However, standard statistical approaches are not suited to identify differentially expressed driver genes on 1p/19q, because hundreds of genes are down-regulated on both chromosomal arms due to the co-deletion making it impossible to distinguish between driver and passenger genes. Further, the recurrence of virtually identical 1p/19q co-deletions in different oligodendrogliomas does not allow to narrow down chromosomal regions on 1p and 19q where driver genes might be located.

Novel computational strategies are required to search for putative cancer candidate genes located within the region of the 1p/19q co-deletion. Generally, the analysis of gene mutations in the context of gene interaction networks represents a promising strategy to address this challenge [24, 39, 65]. Importantly, we recently showed that gene regulatory networks inferred from gene expression and copy number data can be used to quantify impacts of gene copy number mutations on cancer-relevant target genes [64, 65]. The key idea behind this approach is the propagation of gene expression alterations through a gene regulatory network to determine how individual gene copy number mutations influence the expression of other genes in the network. Utilizing such an approach, each individual gene located within the region of the 1p/19q co-deletion can be analyzed offering the unique possibility to search for novel cancer candidate genes that influence the development of oligodendrogliomas.

**Fig. 1:**
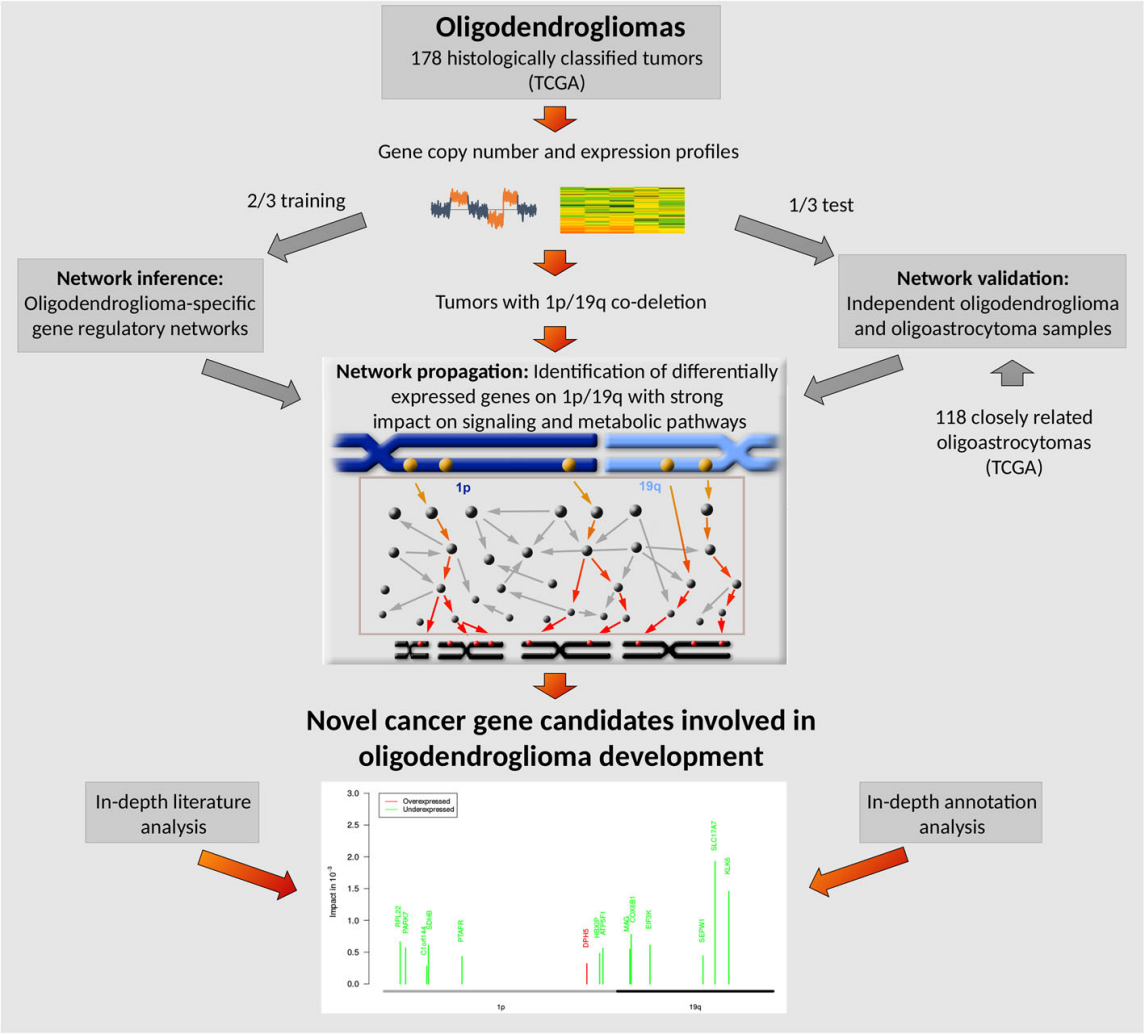
Methodological overview. Gene copy number and expression data of 178 histologically classified oligodendrogliomas from The Cancer Genome Atlas (TCGA) were randomly split into training (two-thirds) and corresponding test (one-third) samples. Oligodendroglioma-specific gene regulatory networks were learned based on gene expression and copy number data of each training set. The obtained networks were validated on their corresponding oligodendroglioma test sets and independent data of closely related oligoastrocytomas from TCGA. Network propagation was applied to genes within the region of the 1p/19q co-deletion to identify those genes that had a strong impact on the expression of known cancer-relevant signaling and metabolic pathways

Here, we develop a network-based approach to identify novel putative cancer gene candidates for oligodendrogliomas (Fig. 1). We utilized gene expression and copy number data of 178 histologically classified oligodendrogliomas from The Cancer Genome Atlas (TCGA) to learn gene regulatory networks. We used these networks to determine impacts of differentially expressed genes with underlying copy number mutations on known cancer-relevant signaling and metabolic pathway genes utilizing network propagation. We screened the region of the recurrent 1p/19q co-deletion and other rarely mutated chromosomal arms revealing several interesting novel putative cancer candidate genes that have the potential to be involved in the development of oligodendrogliomas.

## Materials and methods

### Gene copy number and expression data

DNA copy number profiles (aCGH), gene expression data (RNA-seq), and clinical annotations of 178 histologically classified oligodendrogliomas (133 with and 45 without 1p/19q co-deletion) of the TCGA lower grade glioma (LGG, gdc.cancer.gov) cohort and gene expression data (RNA-seq) of three commercially available normal brain samples (StrataGen, BioChain, and Clonetech) from [38] were considered. The tumors represent the 1p/19q and IDHme subgroups described in [38]. Gene copy number profiles of individual tumors were determined from aCGH profiles as described in [65]. Gene expression counts of tumor and normal samples were jointly normalized with the cyclic loess method using the R function voom of the limma package [61]. We finally included gene copy number and gene expression measurements of 12,285 genes in our data set after excluding all genes with very low expression values (less than 1 read count per million reads mapped) in at least 50% of samples. Further, aCGH and gene expression data of 118 histologically classified oligoastrocytomas (34 with and 84 without 1p/19q co-deletion) of the TCGA LGG cohort were processed in the same way and considered for independent network validation. All processed data are contained in Additional file 1: Table S1 and Additional file 2: Table S2.

### Identification of chromosomal aberrations and gene copy number mutations

Hierarchical clustering of gene copy number profiles of the 178 histologically classified oligodendrogliomas was done using the R function heatmap.2 (euclidean distance, complete linkage) of the R package gplots [79]. We found that 133 tumors had the characteristic 1p/19q co-deletion (Additional file 3: Figure S1, see [38] for all tumors). We considered each of these tumors with 1p/19q co-deletion and determined deleted and duplicated genes. To realize this, we computed the average copy number log2-ratio *r*_*i*_ (*r*_*i*_<0) of tumor to normal DNA within the 1p/19q co-deletion region for tumor *i*∈{1,⋯,133}. We marked each gene in tumor *i* as deleted if its gene-specific copy number log-ratio was less than 0.5·*r*_*i*_. In analogy, we marked each gene as duplicated if its log-ratio was greater than − 0.5·*r*_*i*_. We considered a scaling factor of 0.5 to account for the fact that gene copy number measurements are typically noisy depending on the individual tumor content of the patient samples. This enabled us to specify for each tumor all genes and chromosomal regions (e.g. deletion of 4q, duplication of 7p) affected by deletions or duplications that were visible in the heatmap in addition to the 1p/19q co-deletion (Additional file 3: Figure S1).

### Identification of differentially expressed genes

Differentially expressed genes between oligodendrogliomas with 1p/19q co-deletion and normal brain samples were derived by moderated t-tests using limma’s standard workflow [61]. *P*-values were adjusted for multiple testing by computing *q*-values (R package qvalue) [68]. Under- and overexpressed genes in oligodendrogliomas in comparison to normal brain were selected using a *q*-value cutoff of 0.05 (Additional file 4: Table S3).

### Gene and pathway annotation analysis

Gene annotations (transcription factors/cofactors, kinases, phosphatases, oncogenes, tumor suppressors) and genes included in cancer-relevant signaling and metabolic pathways were obtained from [65]. The number of differentially expressed genes per annotation category was determined separately for under- and overexpressed genes and the significance of gene enrichment in each category was quantified using Fisher’s exact test.

### Inference of gene regulatory networks

Gene expression (log2-ratios of tumor to average normal brain) and gene copy number (log2-ratios of tumor to normal DNA) data of all histologically classified oligodendrogliomas were used to learn gene regulatory networks using the R package regNet [64]. Histologically classified oligodendrogliomas without 1p/19q co-deletion were included to increase the variation of gene expression within the region of the 1p/19q co-deletion to support the selection of relevant links between genes. We randomly divided the oligodendrogliomas into a training set containing two-thirds of the tumors (119) for network inference and a test set containing the remaining one-third of tumors (59) for network validation. For each of the 12,285 genes in our data set, regNet models the expression of each gene as a linear combination of its own gene copy number and the expression of all other genes to determine the most relevant predictors (gene-specific copy number and expression of putative regulators) of each gene [64]. To solve each gene-specific linear model, regNet uses lasso regression [70] in combination with a significance test for lasso [46] to estimate the coefficient and corresponding significance (*q*-value) for each gene-specific predictor. Lasso regression selects the most relevant predictors of each gene and automatically shrinks the coefficients of other irrelevant predictors to zero. To avoid the inclusion of spurious predictors that only represent the local copy number state but not putative regulatory dependencies between genes, we removed local gene-specific predictors 50 genes down- and up-stream of each gene as done in [65]. We finally only considered the most significant predictors of each gene with a q-value equal or less than 0.01. Network inference was very time consuming (390h CPU time per network). Nevertheless, we repeated the genome-wide network inference ten times with different training sets to integrate evidences from different networks into the prediction of novel tumor gene candidates.

### Identification of major regulators

To determine major regulators with many outgoing links to other genes, we defined a scoring scheme that integrates the learned networks. We assume that links that are present in more networks are also more relevant than links that are only found in some networks. First, we counted for each gene *g*∈{1,…,12285} the number of outgoing links *c*_*gi*_ that were observed in *i*∈{1,…,10} of the 10 networks resulting in a count matrix *C*:=(*c*_*gi*_). Next, we standardized each column sum of *C* to 1 to account for different numbers of outgoing links involved in counting. Finally, we determined for each gene its score by summing up the corresponding gene-specific row values of the standardized count matrix *C*. Genes with greater score values have more stable outgoing links across the learned networks than genes with lower scores. This ranking of genes enabled to determine major regulators across the networks and to test if genes of a specific annotation class have greater scores than genes that were not part of this class (Wilcoxon rank sum test).

### Validation of learned networks

To assess the prediction quality of individual gene expression levels by each network, we computed correlations between network-based predicted and experimentally measured gene expression levels for each of the ten networks considering the corresponding network-specific oligodendroglioma test set. We further utilized each network to predict the expression levels of 118 histologically classified oligoastrocytomas (34 with 1p/19q co-deletion, 84 without 1p/19q co-deletion), a tumor type that is closely related to oligodendrogliomas [38, 48]. In addition, to have baseline models for the different validation data sets, we computed 25 random networks (degree-preserving network permutations) for each of the ten learned networks using regNet [64] to compare their prediction quality to those of the ten original networks. To summarize the prediction results of the different networks, we computed median correlations between predicted and measured expression levels and we further analyzed if the obtained median correlation distribution of the original networks was significantly shifted into the positive range compared to the correlation distribution of the random networks using a Wilcoxon rank sum test.

### Network-based impact quantification of gene copy number mutations on signaling and metabolic pathways

We considered all oligodendrogliomas with 1p/19q co-deletion to analyze how differentially expressed genes between tumor and normal brain tissue located within the region of the 1p/19q co-deletion impact on cancer-relevant signaling and metabolic pathways. We used the network propagation algorithm implemented in regNet [64] to realize this. This algorithm considers a learned network and the prediction quality of individual genes to compute direct and indirect impacts between each pair of genes considering all possible network paths (Fig. 1). We have previously shown that this algorithm can correctly predict downstream impacts of gene perturbation experiments [65].

We first computed the total strength of impacts that flow from a differentially expressed gene located within the region of the 1p/19q co-deletion to individual signaling and metabolic pathway genes for each of the ten learned networks. To compare the obtained impacts to random baseline models, we considered the 25 random network instances computed for each of the ten networks to determine the corresponding average impacts of each differentially expressed gene of the 1p/19q region on all signaling and metabolic genes. We next compared the median impact of each gene under the ten original networks to the corresponding average impacts of this gene under the random networks using a paired one-sided Wilcoxon rank sum test and further corrected for multiple testing by computing *q*-values [68]. We used a paired test to account for the fact that the random networks that belong to each of the ten individual networks were derived by degree-preserving network permutations. We considered a one-sided test because only genes with greater impact obtained under corresponding random models are of interest. We considered differentially expressed genes within the region of the 1p/19q co-deletion as high-impact genes if they had significantly greater impacts on signaling or metabolic pathways than under corresponding random networks using a *q*-value cutoff of 0.05.

Moreover, we also analyzed impacts of differentially expressed genes in chromosomal regions that were much less frequently affected by deletions or duplications in oligodendrogliomas. We specifically focused on aberrations of whole chromosomal arms in addition to the characteristic 1p/19q co-deletion. To account for noisy gene copy number measurements, we defined a chromosomal arm to be mutated if at least 80% of its genes were duplicated or deleted, respectively. To validate the considered mutated chromosomal arms, we compared our predictions to those reported for oligodendrogliomas of the POLA cohort [33] and found that they have been previously described (Table 1). We considered each chromosomal arm that was mutated in at least six oligodendrogliomas with 1p/19q co-deletion and defined a corresponding subcohort of oligodendrogliomas that showed these mutations. We considered each subcohort and computed for all differentially expressed genes located on the mutated chromosomal arm corresponding impacts on signaling and metabolic pathway genes as described above.

**Table 1:** Statistics of rarely mutated chromosomal arms

	Deletions					Duplications		
Chromosomal arm	4q	9q	13q	15q	18q	7p	7q	11q
TCGA: OD II + III	9.8%	4.5%	10.5%	9.0%	15.0%	6.0%	9.0%	4.5%
TCGA: OD III	13.8%	5.2%	10.3%	12.1%	20.7%	6.9%	10.3%	6.9%
POLA: OD III	16.2%	14.7%	5.9%	14.7%	8.8%	4.4%	7.3%	19.1%

Chromosomal arms affected by deletions (4q, 9q, 13q, 15q and 18q) and duplications (7p, 7q and 11q) in subsets of oligodendrogliomas in addition to the characteristic 1p/19q co-deletion. Percentages of affected oligodendrogliomas are shown for the TCGA cohort (TCGA: OD II + III comprised 133 oligodendrogliomas of WHO grades II and III, TCGA: OD III comprised 58 oligodendrogliomas of WHO grade III) and the POLA cohort [33] (POLA: OD III comprised 68 oligodendrogliomas of WHO grade III)

## Results and discussion

### Many under- and several overexpressed genes are observed within the region of the 1p/19q co-deletion

We considered all oligodendrogliomas with 1p/19q co-deletion and compared their gene expression profiles to normal brain references to identify differentially expressed genes. We observed 3,068 (23.8%) under- and 3204 (24.9% of genes) overexpressed genes in oligodendrogliomas (*q*-value < 0.05, Additional file 4: Table S3). Only few strongly underexpressed tumor suppressors (log-ratio <−2: *ANO3* and *CDH1*), but several strongly overexpressed oncogenes (log-ratio > 2: *MYC*, *EGFR*, *PDGFRA*, *PIK3CA*, *PRRX1*, *ASCC3*, *ZNF117*, *CRISPLD1*, *CSMD3*, *ALDH1L2*, *MDGA2*, *TSHR* and *H3F3A*) were among these genes.

Considering chromosomal locations, we found that 524 underexpressed genes (45.2% of genes on 1p/19q) and interestingly also 130 overexpressed genes (11.2% of genes on 1p/19q) were located within the region of the 1p/19q co-deletion. We observed strong underexpression for 74 of the 524 underexpressed genes on 1p/19q (log-ratio <−2). The ten most strongly underexpressed genes on 1p/19q were *LC17A7*, *PRKCG*, *RIMS3*, *KIAA1324*, *AK5*, *SLC6A17*, *CD22*, *HPCA*, *MAG* and *CHD5*. We also observed strong overexpression for 10 of the 130 overexpressed genes on 1p/19q (log-ratio > 2: *SAMD11*, *SLC35E2*, *HES5*, *GRHL3*, *RCC1*, *SPOCD1*, *HFM1*, *DLL3*, *IL4I1* and *CACNG6*). Interestingly, *DLL3* and *HES5* are part of the Notch signaling pathway involved in oligodendrocyte specification [56] restricting cell proliferation and tumor growth in glioma mouse models [22].

**Fig. 2:**
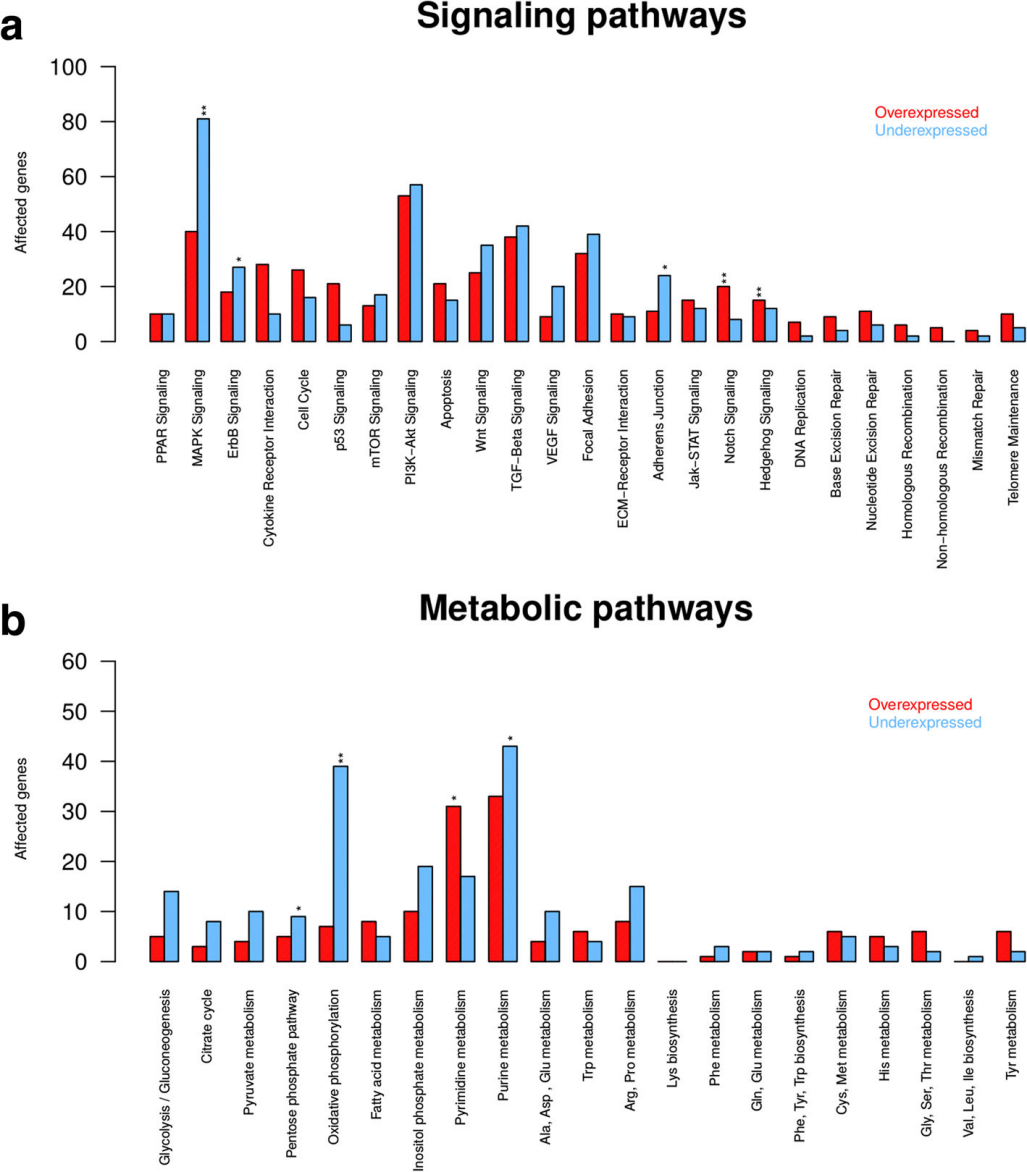
Signaling and metabolic pathway analysis of differentially expressed genes. Differentially expressed genes between oligodendrogliomas and normal brain references (*q*-value < 0.05, Additional file 4: Table S3) were mapped to known cancer-relevant signaling (**a**) and metabolic pathways (**b**). The number of over- and underexpressed genes are shown for each pathway. Asterisks symbols highlight pathways enriched for over- or underexpressed genes (Fisher’s exact test with ’*’ for *P*<0.1 and ’**’ for *P*<0.05)

We further analyzed all differentially expressed genes in the context of known cancer-relevant signaling pathways (Fig. 2). We observed that especially the Notch and Hedgehog signaling were strongly enriched for overexpressed genes, whereas MAPK signaling was enriched for underexpressed genes (Fig. 2a). In addition, also ErbB signaling and the Adherens junction pathway tended to show an enrichment of underexpressed genes. Considering metabolic pathways, we found that the oxidative phosphorylation pathway was enriched for underexpressed genes (Fig. 2b). Also the pyrimidine, purine and pentose phosphate pathway tended to show some enrichment of differentially expressed genes.

### Transcriptional regulatory networks predict tumor gene expression levels

To provide the basis for the impact quantification of gene copy number mutations on cancer-relevant pathways, we used regNet [64] to learn genome-wide transcriptional regulatory networks based on gene copy number and expression data of 178 histologically classified oligodendrogliomas with and without 1p/19q co-deletion. We repeated the genome-wide network inference ten times utilizing different training and test data sets (see “Materials and methods” section for details). The resulting networks had on average 67,900 ± 1080 directed links between regulators and target genes (Additional file 3: Figure S3). More than three quarters of these links were activator links (78%) and the others were inhibitor links.

Next, we integrated the outgoing links of each gene across the ten networks to derive a connectivity score that accounts for the co-occurrence of links (see “Materials and methods” for details). This score is higher for genes with more stable outgoing links across n of networks than for genes with less co-occurring links. We utilized these scores and found that tumor suppressor genes, oncogenes, essential genes and signaling pathway genes had significantly greater connectivity scores than genes that were not included in these categories (Wilcoxon rank sum tests: *P*=0.035 for tumor suppressors, *P*=0.028 for oncogenes, *P*=5.39·10^−9^ for essential genes, *P*=0.01 for signaling pathway genes). The ten genes with the greatest connectivity score were (*GARS*, *CCDC85B*, *NDUFA1*, *SPRED2*, *BIRC6*, *MRPL45*, *EDA2R*, *HMGCS1*, *SLC17A7*, *RAB40B*; Additional file 3: Figure S4). *CCDC85B* is a known downstream target of p53 signaling with reported function as tumor suppressor [26]. Also *SPRED2* is a known tumor suppressor that induces autophagy [32]. *SLC17A7* has been observed as a tumor suppressor in a glioblastoma stem cell line [44]. *BIRC6* can inhibit apoptosis in glioblastoma cell lines [11]. *RAB40B* is a member of the RAS oncogene family potentially involved in the remodeling of the extracellular matrix during invasion of breast cancer [27]. Other genes like *GARS*, *NDUFA1*, *HMGCS1*, and *SLC17A7* have known functions in cellular metabolism. This clearly indicates that major regulators in our networks are known to have important cancer-relevant functions.

**Fig. 3:**
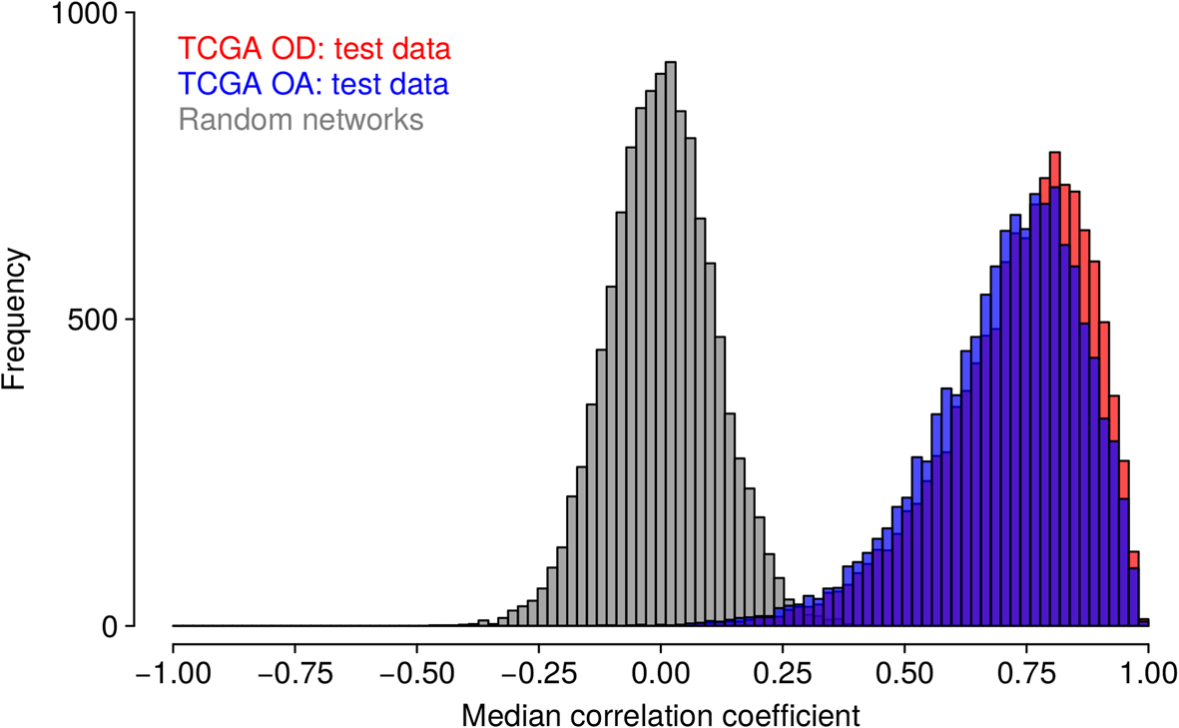
Network-based prediction quality of gene expression levels. The ten learned gene regulatory networks were analyzed for their performance to predict the expression levels of the 12,285 genes in independent tumor test sets (TCGA OD: 59 network-specific oligodendrogliomas left out from network inference, TCGA OA: 118 closely related oligoastrocytomas). Corresponding histograms of gene-specific median correlations between predicted and measured gene expression levels are shown. The strong shift of both histograms (red, blue) into the positive range shows that the prediction quality of oligodendroglioma-specific networks was significantly better than for random networks (grey) of same complexity (Wilcoxon rank sum tests: *P*<2.2·10^−16^)

We further tested the capability of each network to predict the expression level of each of the 12,285 genes in independent oligodendroglioma (59 randomly selected tumors left out from network learning) and closely related oligoastrocytoma (118 samples including 34 tumors with and 84 tumors without 1p/19q co-deletion) test sets that were not considered for network inference. To realize this, we computed correlations between originally measured gene expression levels and corresponding network-based predicted gene expression levels across all tumor samples in each test set for each of the ten networks to analyze the prediction quality. Corresponding median gene-specific correlations integrating the prediction results of the ten networks are summarized in Fig. 3 (see Additional file 3: Figure S5 for individual networks). Overall, the vast majority of genes showed strong positive correlations between measured and predicted expression values with a median correlation of 0.75 for the oligodendroglioma test sets and a median correlation of 0.73 for the oligoastrocytoma test set. We also compared these results to predictions of gene expression levels that were obtained from random networks of same complexity as the originally learned networks (degree-preserving network permutations). We found that our networks made significantly better predictions of originally measured gene expression levels than corresponding random networks (Fig. 3, Wilcoxon rank sum test: *P*<2.2·10^−16^ for each of both test sets).

### Genes directly affected by the 1p/19q co-deletion strongly impact on cancer-relevant signaling pathways

**Fig. 4:**
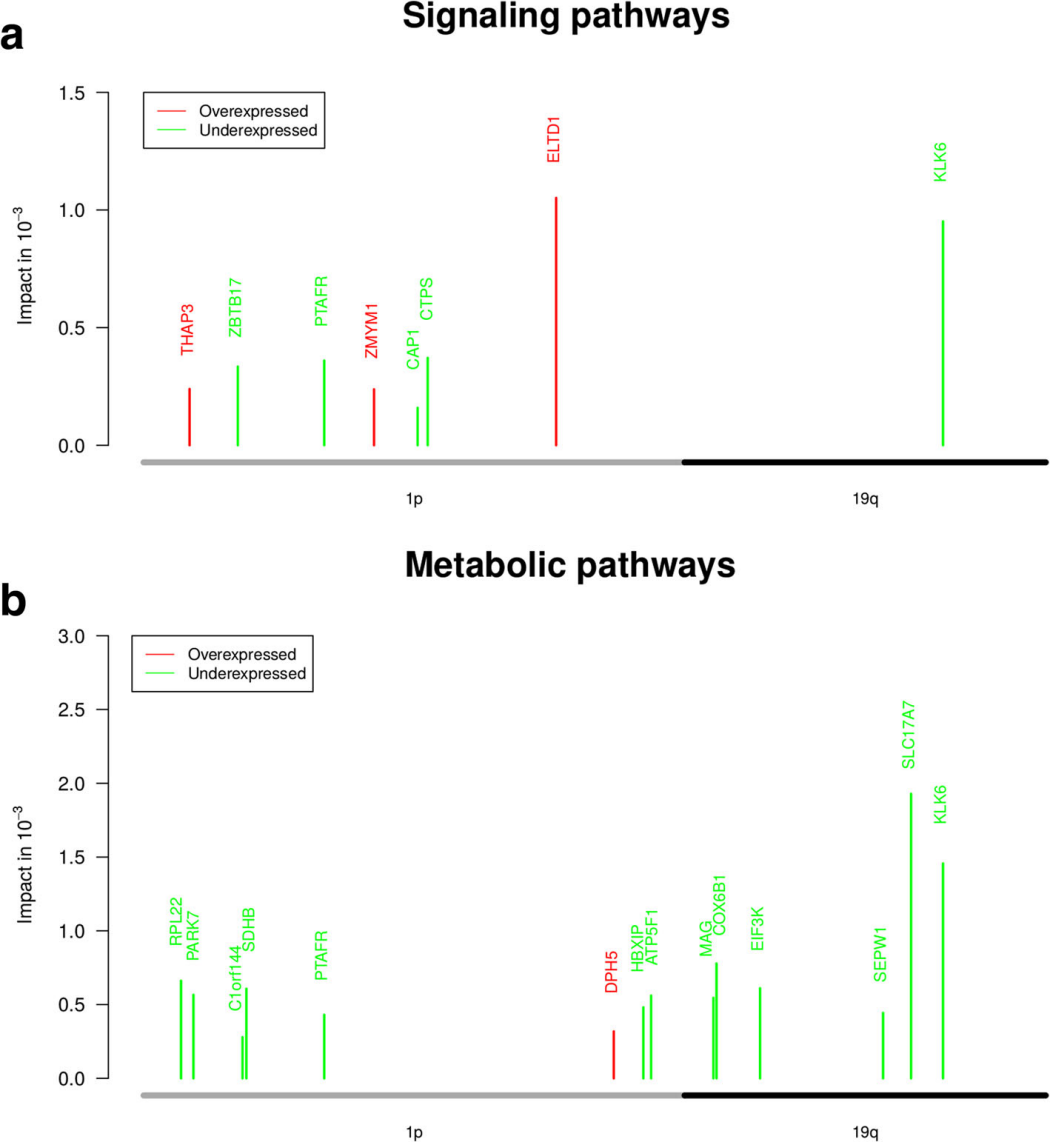
Genes located in the region of the 1p/19q co-deletion with strong impact on signaling and metabolic pathways. Impacts of differentially expressed genes of the 1p/19q region on the expression of known cancer-relevant signaling pathway genes (**a**) and metabolic pathway genes (**b**). All shown genes had significantly greater impacts under the oligodendroglioma-specific gene regulatory networks than under corresponding random networks of same complexity (*q*-value ≤0.05). The high-impact genes are widespread across the region of the 1p/19q co-deletion. Genes colored in green were underexpressed and genes colored in red were overexpressed in oligodendrogliomas compared to normal brain tissue

We utilized the learned networks to determine impacts of differentially expressed genes located within the region of the 1p/19q co-deletion on known cancer-relevant signaling pathway genes (see Fig. 1 for an illustration). To realize this, we considered the 654 differentially expressed genes observed within the 1p/19q region (524 under- and 130 overexpressed genes with *q*-values < 0.05, Additional file 4: Table S3) and applied regNet [64] to compute impacts of these genes on the expression of signaling pathway genes using network propagation. We did this independently for each network and computed corresponding impacts for each gene pair under random networks. We further integrated the scores of the ten networks and determined all differentially expressed 1p/19q-genes with significantly greater impacts on the expression of known cancer signaling pathway genes than under random networks (paired Wilcoxon rank sum tests, *q*-value < 0.05, see “Materials and methods” section for details). Predicted high-impact genes are shown in Fig. 4a and provided in Additional file 5: Table S4. We performed in-depth literature searches and analyzed gene annotations [62] to characterize cellular functions and known cancer-relevant impacts of these genes.

The gene with the greatest impact on signaling pathway genes was *ELTD1* located on the 1p arm. *ELTD1* encodes for a G-protein coupled receptor. The deletion of one copy of the 1p arm in oligodendrogliomas did not lead to a reduced expression of *ELTD1*. We found *ELTD1* significantly overexpressed in oliogodendrogliomas compared to normal brain tissue. *ELTD1* has been identified to represent a key player of tumor angiogenesis [50]. *ELTD1* has also been functionally validated as oncogene in glioblastomas [72, 86]. The microRNA-139-5p has been reported to act as a tumor suppressor inhibiting *ELTD1* expression in glioblastoma cell lines [15].

The only detected high-impact gene located on the 19q arm with strong impact on signaling pathway genes was *KLK6*. *KLK6* encodes for a serine-protease and was strongly underexpressed in oligodendrogliomas compared to normal brain tissue. High expression levels of *KLK6* have been associated with poor prognosis of intracranial tumors [18] and resistance of glioblastomas to cytotoxic agents [19]. *KLK6* has recently been found to be involved in the control of metastasis formation in colon cancer [66].

*PTAFR* located on the 1p arm encodes for a G-protein coupled receptor involved in the regulation of cell proliferation and angiogenesis. *PTAFR* was strongly underexpressed in oligodendrogliomas compared to normal brain. *PTAFR* is a putative oncogene and has been reported to play a role in different types of cancer including the activation of PI3K-Akt signaling in esophageal cancer [12] or the support of prostate cancer development via *ERK1/ERK2* signaling [30].

*ZBTB17* located on the 1p arm was underexpressed in oligodendrogliomas compared to normal brain tissue. *ZBTB17* encodes a transcriptional regulator interacting with *MYC*-genes. Reduced expression of *ZBTB17* due to heterozygous loss of 1p36 has been reported to increase the aggressiveness of neuroblastomas [25]. This suggests that *ZBTB17* is a putative tumor suppressor gene.

*CAP1* located on the 1p arm was underexpressed in oligodendrogliomas compared to normal brain tissue. *CAP1* is involved in the cyclic AMP pathway and interacts with the actin cytoskeleton influencing cell adhesion [82]. *CAP1* expression has been reported to be positively correlated with proliferation, migration, invasion, and WHO grade of gliomas [3, 21].

So far, no roles in cancer have been reported for the two overexpressed high-impact genes *THAP3* and *ZMYM1* located on the 1p arm. Both genes are likely to encode transcription factors. *THAP3* is involved in the regulation of cell proliferation [51]. *ZMYM1* could be involved in the regulation of the cytoskeletal organization and cell morphology.

Further, we used our network propagation algorithm to predict potential regulatory downstream effects of high-impact genes on individual cancer-relevant signaling pathways (Additional file 3: Figure S6a). Especially the overexpression of *ELTD1* and the underexpression of *PTAFR* in oligodendrogliomas tend to influence the expression of several signaling pathways suggesting complex regulatory dependencies that support or counteract oligodendroglioma growth. Specific impacts of individual high-impact genes are summarized in Additional file 3: Texts S1.

In summary, depending on the expression states in combination with reported roles in cancer, genes like *ELTD1*, *ZBTB17*, or *THAP3* are likely to support oligodendroglioma growth, whereas other genes like *KLK6*, *PTAFR*, or *CAP1* may restrict the speed of tumor growth. This might contribute to the overall better prognosis of oligodendroglioma patients in comparison to patients with other gliomas [69].

### Genes directly affected by the 1p/19q co-deletion strongly impact on metabolic pathways

Similar to the analysis of signaling pathways, we used network propagation to identify those differentially expressed genes within the region of the 1p/19q co-deletion that had strong impacts on the expression of metabolic pathway genes. We predicted 14 high-impact genes widespread across the 1p/19q region (*q*-value < 0.05, Fig. 4b, Additional file 6: Table S5). All genes were underexpressed in oligodendrogliomas compared to normal brain, except for overexpressed *DPH5*. Two genes with strong impact on signaling pathways (*KLK6*, *PTAFR*) were also among these high-impact genes. In contrast to our previous impact quantification for signaling pathways that revealed only one high-impact gene on the 19q arm (Fig. 4a), we now found six genes (*MAG*, *COX6B1*, *EIF3K*, *SEPW1*, *SLC17A7*, *KLK6*) with strong impact on the expression of metabolic pathway genes on 19q (Fig. 4b). We again performed in-depth gene annotation analyses and literature searches to summarize known functions and roles in cancer.

*SLC17A7*, the gene with the greatest impact on the expression of metabolic pathways, has been observed as tumor suppressor in a glioblastoma stem cell line [44]. *SLC17A7* is located on the 19q arm, encodes for a vesicle-bound, sodium-dependent phosphate transporter expressed in neuron-rich regions, and was strongly underexpressed in oligodendrogliomas compared to normal brain.

*SDHB* is located on the 1p arm, encodes for the succinate dehydrogenase complex subunit B, and was underexpressed in oligodendrogliomas in comparison to normal brain. Germline mutations of *SDHB* have been reported for patients with head and neck paraganglioma [5] and phaeochromocytomas [7]. Succinate accumulated in *SDHB*-mutated cells inhibits alpha-ketoglutarate-dependent enzymes leading to the activation of hypoxia induced genes and hypermethylation of DNA and histones in paraganglioma [4, 40]. Similarly, a knockdown of *SDHB* in mouse ovarian cancer cells enhanced cell proliferation and induced hypermethylation of histones promoting an epithelial-to-mesenchymal transition [2]. All these findings suggest that reduced expression of *SDHB* in oligodendrogliomas may support and possibly enhance the epigenetic reprogramming via the same pathomechanism induced by a heterozygous *IDH*-mutation that is found in each oligodendroglioma [13, 48].

*PARK7* located on the 1p arm was underexpressed in oligodendrogliomas compared to normal brain tissue. *PARK7* encodes for a peptidase that protects cells against oxidative stress. Downregulation of *PARK7* has been associated with a reduction of cell proliferation, migration, and invasion of glioma cell lines [31]. Downregulation of *PARK7* in clear renal cell carcinoma cells increased cisplatin-induced apoptosis [73]. *PARK7* has been reported as oncogene in different cancers activating PI3K-Akt, MAPK, and mTOR signaling to protect cells against hypoxic stress [77].

*HBXIP* located on the 1p arm was underexpressed in oligodendrogliomas compared to normal brain tissue. *HBXIP* functions as a cofactor of survivin in the suppression of apoptosis [49]. *HBXIP* has been reported to promote the proliferation and migration of breast cancer cells [45]. Conversely, suppression of *HBXIP* has been found to reduce cell proliferation, migration and invasion of bladder carcinomas [42]. This suggests that the underexpression of *HBXIP* could counteract oligodendroglioma growth.

*SEPW1* is located on 19q, encodes for a selenoprotein that functions as an glutathione antioxidant, and was underexpressed in oligodendrogliomas compared to normal brain. *SEPW1* has been mapped to a putative tumor suppressor region on the 19q arm of gliomas [67]. *SEPW1* has been shown to be involved in the control of cell cycle progression [23] and to regulate expression, activation and degradation of *EGFR* [1].

*C1orf144* (*SZRD1*) located on 1p was underexpressed in oligodendrogliomas in comparison to normal brain tissue. *C1orf144* has recently been reported as a potential tumor suppressor in cervical cancer involved in the regulation of cell cycle arrest in G2 and induction of apoptosis [84].

*MAG* located on 19q was underexpressed in oligodendrogliomas compared to normal brain tissue. *MAG* encodes for a membrane protein involved in myelination of oligodendrocytes, protection of neurons against apoptosis, and inhibition of neurite outgrowth [59].

Further, only *DPH5* located on 1p was overexpressed in oligodendrogliomas compared to normal brain. *DPH5* encodes for a specific methionine-dependent methyltransferase involved in diphthamide synthesis. Diphthamide, a post-transcriptionally modified histidine, is required for eEF-2, which is essential for protein biosynthesis. Further, two underexpressed high-impact genes, *RPL22* and *EIF3K*, known to be important for protein synthesis were found. Strong impacts of genes involved in protein synthesis might represent a byproduct of increased transcription in tumors. In addition, *COX6B1* located on 19q and *ATP5F1* located on 1p were underexpressed in oligodendrogliomas in comparison to normal brain. Both genes have functions in the respiratory chain.

In addition, we also used our network propagation algorithm to further predict potential regulatory downstream effects of high-impact genes from Fig. 4b on individual metabolic pathways (Additional file 3: Figure S6b). Interestingly, six genes were predicted to contribute to a downregulation of the oxidative phosphorylation. Detailed information to specific impacts of individual high-impact genes are summarized in Additional file 3: Text S1.

Again, genes like *SLC17A7*, *SDHB*, *SEPW1*, or *SZRD1* may support oligodendroglioma growth and other genes like *PARK7* or *HBXIP* may restrict the speed of tumor growth. Such counteracting impacts could contribute to a better prognosis [69].

### Impact of rare gene copy number mutations on cancer-relevant signaling and metabolic pathways

**Fig. 5:**
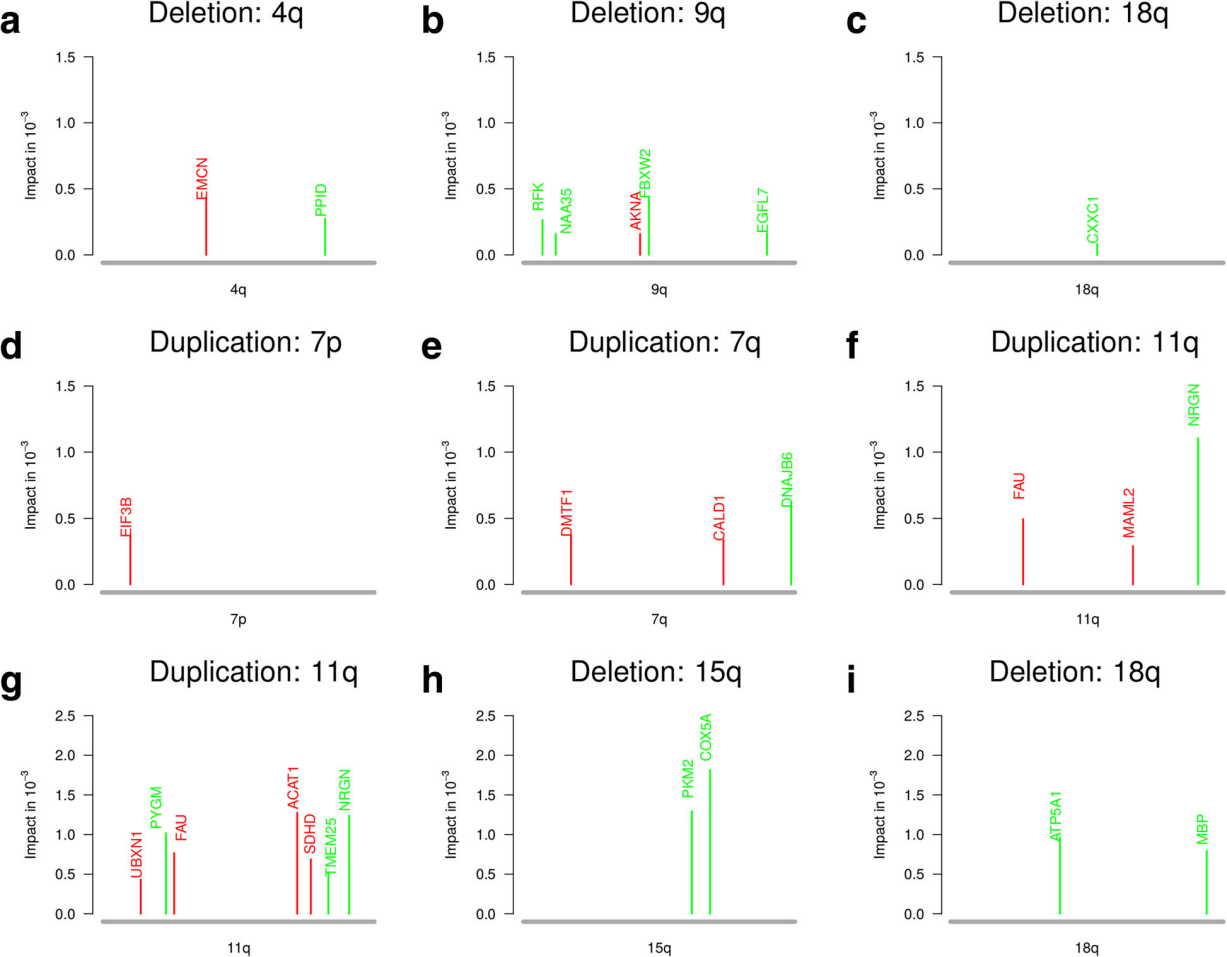
Genes located on rarely mutated chromosomal arms with strong impact on signaling and metabolic pathways. Impacts of differentially expressed genes of rarely mutated chromosomal arms (Table 1) on the expression of known cancer-relevant signaling pathway genes (**a**–**f**) and metabolic pathway genes (**g**–**i**). All shown genes had significantly greater impacts under the oligodendroglioma-specific gene regulatory networks than under corresponding random networks of same complexity (*q*-value ≤0.1). Genes colored in green were underexpressed and genes colored in red were overexpressed in oligodendrogliomas with the corresponding chromosomal arm mutation in comparison to normal brain tissue

We further used our network-based impact quantification strategy to determine if potential candidate genes with high-impact on signaling or metabolic pathways are located on chromosomal arms that were rarely affected by deletions or duplications in oligodendrogliomas with 1p/19q co-deletion (Table 1; deletions: 4q, 9q, 13q, 15q, 18q; duplications: 7p, 7q, 11q; Additional file 3: Figure S1). All these additional mutations have previously been observed in the POLA cohort [33] and several of these mutations were also observed in copy number profiles of single oligodendroglioma cells [71]. These additional copy number mutations occurred more frequently in oligodendrogliomas of WHO grade III than in grade II tumors suggesting that they are associated with tumor progression and may impact on survival [35, 74]. See Additional file 3: Text S2 for further details to subgroups of oligodendrogliomas with additional chromosomal arm mutations. We first determined for each subcohort of oligodendrogliomas with a specific chromosomal arm mutation all differentially expressed genes in comparison to normal brain tissue (*q*-value ≤0.05). We next analyzed all differentially expressed genes of a mutated chromosomal arm to identify those genes that were predicted to have a strong impact on the expression of cancer-relevant signaling (Additional file 7: Table S6) and metabolic pathways (Additional file 8: Table S7) utilizing network propagation. We predicted 15 differentially expressed genes with strong impact on signaling pathways on the chromosomal arms 4q, 9q, 7p, 7q, 11q, and 18q (Fig. 5a–f) and 12 genes with strong impact on metabolic pathways on the chromosomal arms 7p, 11q, 15q, and 18q (Fig. 5g–i, 7p not shown) at a *q*-value cutoff of 0.1 (less stringent than before because of much smaller sample sizes). Functional annotations and literature searches of all predicted high-impact genes are summarized in Additional file 3: Text S3 for signaling pathways and in Additional file 3: Text S4 for metabolic pathways. Next, we only briefly highlight some findings.

Considering genes with high-impact on signaling pathways (Fig. 5a–f), we identified several overexpressed genes in subcohorts of oligodendrogliomas with additional chromosomal arm mutations that were previously found to be involved in tumorigenesis. For example, *EMCN* located on the q-arm of chromosome 4 was overexpressed in oligodendrogliomas with 4q deletion. *EMCN* encodes a glycoprotein that can inhibit adhesion of cells to the extracellular matrix [34]. *EIF3B* located on the p-arm of chromosome 7 was overexpressed in oligodendrogliomas with 7p duplication. *EIF3B* encodes a subunit of the eukaryotic translation initiation factor. A knockdown of *EIF3B* inhibited cell proliferation and increased apoptosis in a glioblastoma cell line [43]. *CALD1* located on the q-arm of chromosome 7 was overexpressed in oligodendrogliomas with 7q duplication. *CALD1* is involved in the regulation of the neovascularization of gliomas [85] and has been associated with tamoxifen resistance of breast cancer [17]. Also *DNAJB6* located on the q-arm of chromosome 7 was overexpressed in oligodendrogliomas with 7q duplication. Overexpression of *DNAJB6* has been reported to promote invasion of colorectal cancer [83]. In addition to these putative oncogenes, we also observed two overexpressed genes with potential tumor suppressor functions that may counteract oligodendroglioma development. *DMTF1* located on 7q encodes a transcription factor with a cyclin D-binding domain that has been shown to inhibit cell growth and cell cycle progression in bladder cancer [57]. Further, *FAU* located on 11q encodes a fusion protein that has been reported to be involved in the regulation of apoptosis of breast cancer [58].

Considering genes with high-impact on metabolic pathways (Fig. 5g–i), we identified four underexpressed genes in subcohorts of oligodendrogliomas with additional chromosomal arm mutations with functions in cellular energy metabolism and impacts on cell migration, apoptosis, or blood vessel development in cancer (deletion of 15q: *COX5A*, *PKM2*; deletion of 18q: *ATP5A1*; duplication of 11q: *PYGM*; Additional file 3: Text S4). In addition, *UBXN1* located on the q-arm of chromosome 11 was overexpressed in oligodendrogliomas. *UBXN1* encodes a ubiquitin-binding protein and has been reported to inhibit the tumor suppressor *BRAC1* [81]. Interestingly, we found that *SDHD* located on 11q was overexpressed in oligodendrogliomas with 11q duplication. This might represent a response to the reduced expression of *SDHB* discussed before. Further, activation of the expression of the tumor suppressor *CDKN1A* in response to the loss of *SDHD* expression has been reported [52]. Thus, overexpressed *SDHD* might counteract the expression of *CDKN1A* to support cell proliferation. Moreover, also *ACAT1* located on 11q was overexpressed. *ACAT1* encodes a mitochondrially localized acetyl-CoA acetyltransferase. Inhibition of *ACAT1* by Avasimibe inhibited cell growth by inducing cell cycle arrest and apoptosis in glioblastoma cell lines [6, 55]. Further, inhibition of *ACAT1* has also been shown to suppress growth and metastasis of pancreatic cancer [41].

### In-depth analysis of known potential tumor suppressor genes *FUBP1* and *CIC*

We also performed a detailed analysis of the expression behavior and corresponding network-based impacts of the potential tumor suppressors *FUBP1* and *CIC* reported for oligodendrogliomas [8]. *FUBP1* located on 1p and *CIC* located on 19q were both moderately underexpressed in oligodendrogliomas with 1p/19q co-deletion compared to normal brain references (Additional file 4: Table S3). Further, oligodendrogliomas with additional small deletions, insertions or point mutations within *FUBP1* or *CIC* showed moderately reduced expression of these genes compared to oligodendrogliomas without mutations. This trend was much stronger for tumors with *FUBP1* mutations (average expression 4.58 vs. 5.25 comparing 38 tumors with to 95 tumors without mutation, t-test: *P*=0.0001) than for tumors with *CIC* mutations (average expression 6.42 vs. 6.60 comparing 85 tumors with to 48 tumors without mutation, t-test: *P*=0.01).

Further, *FUBP1* has been reported to negatively regulate the expression of *MYC* [8]. This relationship was also predicted by our network propagation approach. *FUBP1* had a stronger impact on *MYC* comparing our networks to corresponding random networks (paired Wilcoxon rank test: *P*<0.001, see “Materials and methods” for details). Globally, *FUBP1* and *CIC* underexpression had moderate impacts on different signaling and metabolic pathways (Additional file 3: Figure S7). Thus, reduced expression of both genes due to the 1p/19q co-deletion could contribute to tumor development, but both genes were not among the predicted putative high impact genes with altered gene expression levels. Still, other pathomechanisms triggered by small deletions, insertions or point mutations within *FUBP1* or *CIC* could play an important role in affected tumors.

## Conclusions

The clinical relevance of the 1p/19q co-deletion has been known for many years, but detailed insights to underlying pathomechanisms are not known. Our computational approach provides a novel starting point to characterize molecular changes induced by the 1p/19q co-deletion. We predicted several interesting cancer candidate genes widespread across the region of the 1p/19q co-deletion with strong impact on signaling and metabolic pathways. These candidate genes are possibly involved in the development of oligodendrogliomas. Interestingly, several of these genes (e.g. *ELTD1*, *SDHB*, *SEPW1*, *SLC17A7*, *SZRD1*, *THAP3*, *ZBTB17*) are likely to push, whereas other genes (e.g. *CAP1*, *HBXIP*, *KLK6*, *PARK7*, *PTAFR*) might restrict oligodendroglioma development. This observation could contribute to the fact that oligodendrogliomas have an improved prognosis in comparison to other types of gliomas. Importantly, the overexpression of *ELTD1* in oligodendrogliomas despite the loss of 1p indicates that this gene may act as oncogene as reported for closely related glioblastomas. Similarly, the underexpression of *SLC17A7* in oligodendrogliomas may counteract its known function as tumor suppressor reported for glioblastomas. Moreover, the underexpression of *SDHB* may contribute to the epigenetic reprogramming of oligodendrogliomas via the same pathomechanism as triggered by the *IDH*-mutation. All these findings indicate that several genes located on 1p/19q may simultaneously influence tumor development. Further, we also predicted cancer candidate genes on rarely mutated chromosomal arms that are likely to contribute to oligodendroglioma development and tumor progression in subcohorts of patients. In sum, our computational predictions contribute to a better understanding of the pathology of the 1p/19q co-deletion, might open opportunities for novel experimental studies, and possibly trigger ideas for the development of targeted treatment strategies.

## Additional files

Additional file 1 **Table S1.** Gene copy number data. (XLS 68300 kb)

Additional file 2 **Table S2.** Gene expression data. (XLS 48300 kb)

Additional file 3 **Texts S1–S4** and **Figures S1–S7**. (PDF 1800 kb)

Additional file 4 **Table S3.** Differentially expressed genes between oligodendrogliomas with 1p/19q co-deletion and normal brain tissue. (XLS 2240 kb)

Additional file 5 **Table S4.** Genes located on 1p/19q with strong impact on signaling pathways. (XLS 115 kb)

Additional file 6 **Table S5.** Genes located on 1p/19q with strong impact on metabolic pathways. (XLS 115 kb)

Additional file 7 **Table S6.** Genes located on rarely mutated chromosomal arms with strong impact on signaling pathways. (XLS 187 kb)

Additional file 8 **Table S7.** Genes located on rarely mutated chromosomal arms with strong impact on metabolic pathways. (XLS 187 kb)

## Abbreviations

1p: p-arm of chromosome 1
19q: q-arm of chromosome 19
OD: oligodendroglioma
TCGA: The cancer genome atlas
WHO: World health organization
