# Supplementary material for: Network-based analysis of oligodendrogliomas predicts novel cancer gene candidates within the region of the 1p/19q co-deletion
Source: Acta Neuropathol Commun. 2018 Jun 11;6:49. doi: 10.1186/s40478-018-0544-y (PMC5996550; doi:10.1186/s40478-018-0544-y)
Supplement: Supplementary file 3 — Texts S1–S4 and Figures S1–S7. (PDF 1800 kb) [file 40478_2018_544_MOESM3_ESM.pdf]

Network-based analysis of oligodendrogliomas predicts novel cancer gene candidates within the region of the 1p/19q co-deletion

Supplementary Texts and Figures

Josef Gladitz[1], Barbara Klink[2,3] and Michael Seifert[1,3]

[1]Carl Gustav Carus Faculty of Medicine, Technische Universität Dresden, Institute for Medical Informatics and Biometry (IMB), Fetscherstr. 74, D-01307, Dresden, Germany; [2]Institute for Clinical Genetics, Carl Gustav Carus Faculty of Medicine, Technische Universität Dresden, Dresden, Germany; [3]National Center for Tumor Diseases (NCT), Dresden, Germany

Contact: michael.seifert@tu-dresden.de

Contents

1 Text S1: Predicted downstream effects of high-impact genes on individual signaling and metabolic pathways 2

1.1 Prediction of downstream effects of high-impact genes on individual cancer signaling pathways . . . 2
1.2 Prediction of downstream effects of high-impact genes on individual metabolic pathways . 2

2 Text S2: Subgroups of oligodendrogliomas show additional gene copy number mutations 3

3 Text S3: Impact of rare gene copy number mutations on signaling pathways 4

3.1 Differentially expressed genes directly affected by rare chromosomal arm mutations with strong impact on signaling pathways . . . 4
3.2 Chromosome 4: q-arm deletion in 13 of 133 oligodendrogliomas . . . 4
3.3 Chromosome 9: q-arm deletion in 6 of 133 oligodendrogliomas . . . 5
3.4 Chromosome 18: q-arm deletion in 20 of 133 oligodendrogliomas . . . 5
3.5 Chromosome 7: p-arm duplication in 8 of 133 oligodendrogliomas . . . 5
3.6 Chromosome 7: q-arm duplication in 12 of 133 oligodendrogliomas . . . 5
3.7 Chromosome 11: q-arm duplication in 6 of 133 oligodendrogliomas . . . 6

4 Text S4: Impact of rare gene copy number mutations on metabolic pathways 6

4.1 Differentially expressed genes directly affected by rare chromosomal arm mutations with strong impact on metabolic pathways . . . 7
4.2 Chromosome 15: q-arm deletion in 12 of 133 oligodendrogliomas . . . 7
4.3 Chromosome 18: q-arm deletion in 20 of 133 oligodendrogliomas . . . 7
4.4 Chromosome 7: p-arm duplication in 8 of 133 oligodendrogliomas . . . 7
4.5 Chromosome 11: q-arm duplication in 6 of 133 oligodendrogliomas . . . 8

5 Supporting figures 9

5.1 Figure S1: Hierarchical clustering of histologically classified oligodendrogliomas based on gene copy number data . . . 9
5.2 Figure S2: Survival analysis of oligodendrogliomas with 1p/19q co-deletion . . . 10
5.3 Figure S3: Learned gene regulatory networks . . . 11
5.4 Figure S4: Genes with many outgoing links in learned networks . . . 12
5.5 Figure S5: Gene expression prediction quality of individual networks . . . 13
5.6 Figure S6: Downstream effects of high-impact genes on individual cancer-relevant signaling and metabolic pathways . . . 14

5.7 Figure S7: Impacts of FUBP1 and CIC underexpression on cancer-relevant signaling and metabolic pathways 15

1 Text S1: Predicted downstream effects of high-impact genes on individual signaling and metabolic pathways

We also determined how the expression states of the previously predicted high-impact genes of Fig. 4 of the main manuscript putatively influence the expression of specific signaling or metabolic pathways. To realize this, we used the network propagation algorithm of regNet [1] to quantify putative inhibitory (negative) or activating (positive) impacts of each high-impact gene on individual signaling and metabolic pathways (regNet: relative impacts). In-depth details to the underlying computations are provided in the appendix of [1]. Since expression profiles of oligodendrogliomas were very similar, we computed these impacts for the median expression profile of all 1p/19q co-deleted tumors. The two following sections summarize the predicted regulatory impacts of high-impact genes on individual cancer-relevant signaling and metabolic pathways.

1.1 Prediction of downstream effects of high-impact genes on individual cancer signaling pathways

We used network propagation [1] to predict how the previously described high-impact genes (see Fig. 4a of main manuscript) putatively influence the expression of genes of individual cancer-relevant signaling pathways (Fig. S6a). Generally, we observed three groups of genes (i) *ELTD1* and *ZMYM1* that activate specific pathways, (ii) *KLK6* and *THAP3* that inhibit and activate different pathways, and (iii) *CTPS*, *CAP1*, *ZBTB17*, and *PTAFR* that inhibit specific pathways. Similar to reported oncogenic functions [2–4], we predicted that overexpressed *ELTD1* in oligodendrogliomas may positively regulate the expression of genes of several pathways (ECM-Receptor interaction, MAPK signaling, PI3K-Akt signaling, Adherens junction, Notch signaling, Focal adhesion). Further, genes of the PI3K-Akt signaling pathway were also predicted to be positively regulated by overexpressed *ZMYM1* and underexpressed *KLK6*. We also predicted that overexpressed *THAP3* may positively regulate the expression of cell cycle genes and negatively regulate the expression of DNA base excision repair genes. Underexpressed *CTPS* may negatively regulate the expression of cell cycle and homologous DNA repair genes. Further, underexpressed *ZBTB17* may negatively regulate the expression of base excision repair, Notch signaling and Wnt signaling genes. Underexpressed *CAP1* may negatively regulate the expression of adherens junction and VEGF signaling genes. Moreover, we predicted that underexpressed *PTAFR* may negatively regulate the expression of genes of several signaling pathways (PI3K-Akt signaling, Focal adhesion, Cytokine receptor signaling, TGF-Beta signaling, VEGF signaling, JAK-STAT signaling, ErbB signaling, Apoptosis, mTOR signaling). This is in good agreement with its annotated functions [5], but the putative role of *PTAFR* in oligodendroglioma development is complex. Oncogenic functions of *PTAFR* activating PI3K-Akt signaling were reported for esophageal cancer [6]. Our predictions indicate that the negative regulation of PI3K-Akt signaling by *PTAFR* could partly counteract cellular process supporting tumor development, whereas the negative regulation of pathways such as apoptosis could support the development of oligodendrogliomas.

1.2 Prediction of downstream effects of high-impact genes on individual metabolic pathways

We again used network propagation [1] to predict how previously described high-impact genes of Fig. 4b of the main manuscript putatively influence the expression of genes of specific metabolic pathways

(Fig. S6b). Interestingly, we found that the underexpression of six genes (*ATP5F1*, *PARK7*, *EIF3K*, *SEPW1*, *SDHB*, *COX6B1*) in oligodendrogliomas compared to normal brain tissue led to a downregulation of the expression of genes of the oxidative phosphorylation pathway, whereas only the underexpression of *HBXIP* was predicted to activate the expression of this pathway. The downregulation of the oxidative phosphorylation pathway has been reported for many human carcinomas [7]. In accordance with the Warburg effect [8], this downregulation is known to lead to a switch from oxidative phosphorylation as main source of energy production to glycolysis to adapt to anaerobic conditions, to account for disturbance of mitochondrial energy production, and to enable rapid energy production for tumor growth [9]. Further, underexpressed *SDHB* was predicted to activate the expression of genes of the citrate cycle, whereas *ATP5F1* was predicted to inhibit the expression of this pathway. The disturbance of the citrate cycle by *SDHB* deficiency has been reported for pheochromocytoma and paraganglioma leading to increased cellular succinate levels [10]. Increased succinate levels can inhibit alpha-ketoglutarate-dependent enzymes [11], which may contribute to known epigenetic alterations observed for oligodendrogliomas due to the presence of an *IDH*-mutation [12,13]. Underexpressed *PTAFR* was predicted to downregulate genes of the inositol phosphate metabolism. Underexpressed *RPL22* was predicted to activate the purine metabolism and to downregulate the pentose phosphate pathway. Overexpressed *DPH5* was predicted to activate the expression of the purine metabolism and the pentose phosphate pathway.

2 Text S2: Subgroups of oligodendrogliomas show additional gene copy number mutations

We used hierarchical clustering to group the gene copy number profiles of the 133 histologically classified oligodendrogliomas with 1p/19q co-deletion from TCGA. We observed several less frequently occurring recurrent deletions (4q, 9q, 13q, 15q, 18q) and duplications (7p, 7q, 11q) affecting chromosomal arms of subgroups of these tumors (Fig. S1). The majority of these additional copy number mutations was observed for oligodendrogliomas of WHO grade III, but also several WHO grade II tumors had additional copy number mutations besides the 1p/19q co-deletion (median number of additional gene copy number mutations: 113.5 for OD II vs. 247.5 for OD III, Wilcoxon rank sum test, $P = 0.008$).

We tested if oligodendroglioma patients of WHO grade II and III with additional chromosomal arm mutations differ in survival from oligodendroglioma patients of WHO grade II and III that only showed a 1p/19q co-deletion, but we did not find a significant survival difference between both patient groups (log-rank test: $P = 0.37$, 55 patients with additional chromosomal arm mutations vs. 78 patients with 1p/19q co-deletion only, Fig. S2). Nevertheless, it is still likely that potential driver genes are also located in some of these additionally mutated regions. Since these were rare events, the sample sizes are too small to evaluate each individual region separately.

We further compared the additionally mutated chromosomal arms to chromosomal mutations reported for oligodendrogliomas of WHO grade III from the POLA cohort [14]. Despite of differences in observed mutation frequencies, we found that all mutations of chromosomal arms that we revealed for oligodendrogliomas of the TCGA cohort have also been observed in the POLA cohort (see Tab. 1 in main manuscript). This indicates that these chromosomal mutations did not occur at random, but rather represent less frequent recurrent mutational patterns that may influence the development of some oligodendrogliomas. Further, several of these mutated chromosomal arms were also observed in copy number profiles of single oligodendroglioma cells (e.g. deletions of chromosome 4 and 13 and duplications of chromosome 7 and 11) [15].

3 Text S3: Impact of rare gene copy number mutations on signaling pathways

In the main manuscript, we have analyzed all differentially expressed genes located within the region of the 1p/19q co-deletion to identify genes with strong impact on the expression of signaling and metabolic pathways. The 1p/19q co-deletion was present in each of the 133 analyzed oligodendrogliomas (Fig. S1). Our utilized network-based impact quantification strategy further enabled the identification of potential tumor gene candidates within chromosomal regions that were recurrently observed in smaller groups of oligodendrogliomas (see Tab. 1 of main manuscript; deletions: 4q, 9q, 13q, 15q, 18q; duplications: 7p, 7q, 11q). We first determined for each subset of oligodendrogliomas with a specific chromosomal arm mutation all differentially expressed genes in comparison to normal brain tissue (q-value ≤ 0.05, see main manuscript for general details to methods used for gene expression analysis). We next analyzed all differentially expressed genes of each mutated chromosomal arm with our network-based impact quantification strategy to identify differentially expressed genes within these regions that had a strong impact on the expression of cancer-relevant signaling pathway genes (Tab. S6, paired Wilcoxon rank sum tests, q-value ≤ 0.1, see main manuscript for general details to impact computations). We were able to identify putative high-impact tumor candidate genes on 4q, 9q, 7p, 7q, 11q, and 18q. We again performed in-depth literature searches and analyzed gene annotations to characterize potential cancer-relevant functions of these genes. In the following, we summarize our findings for individual high-impact candidate genes identified on the different chromosomal arms.

3.1 Differentially expressed genes directly affected by rare chromosomal arm mutations with strong impact on signaling pathways

Gene	Location	Gene expression	Impact in 10^{-3}
EMCN	4q (loss)	1.10	0.4
PPID	4q (loss)	-1.23	0.2
RFK	9q (loss)	-2.00	0.3
NAA35	9q (loss)	-0.26	0.2
AKNA	9q (loss)	0.27	0.2
FBXW2	9q (loss)	-1.39	0.4
EGFL7	9q (loss)	-1.12	0.2
CXXC1	18q (loss)	-0.12	0.1
EIF3B	7p (gain)	1.27	0.4
DMTF1	7q (gain)	1.21	0.4
CALD1	7q (gain)	1.08	0.3
DNAJB6	7q (gain)	-1.61	0.6
FAU	11q (gain)	1.51	0.5
MAML2	11q (gain)	3.93	0.3
NRGN	11q (gain)	-5.28	1.1

Predicted high-impact genes located on chromosomal arms rarely mutated in some oligodendrogliomas (Tab. S6, q-value ≤ 0.1). Gene expression: average $\log_2$-ratio of tumor to normal. loss: deletion of chromosomal arm. gain: duplication of chromosomal arm.

3.2 Chromosome 4: q-arm deletion in 13 of 133 oligodendrogliomas

The loss of the q-arm of chromosome 4 did not lead to underexpression of *EMCN*. We found that *EMCN* was overexpressed in oligodendrogliomas with 4q deletion compared to normal brain tissue. *EMCN* encodes a specific glycoprotein that has been found to inhibit cell adhesion to the extracellular matrix

[16]. Further, *PPID* was underexpressed in the oligodendrogliomas with 4q deletion. *PPID* encodes an isomerase that assists in protein folding.

3.3 Chromosome 9: q-arm deletion in 6 of 133 oligodendrogliomas

RFK was underexpressed in oligodendrogliomas with 9q deletion in comparison to normal brain tissue. *RFK* encodes a riboflavin kinase that converts riboflavin (vitamin B2) into flavin mononucleotide and flavin adenine dinucleotide (FAD), which are essential cofactors of dehydrogenases, reductases, and oxidases. *RFK* overexpression has been reported to protect prostate cancer cells against oxidative stress and cisplatin [17]. *NAA35* was weakly underexpressed in oligodendrogliomas with 9q deletion in comparison to normal brain tissue. *NAA35* encodes an acetyltransferase. Functional annotations suggest that *NAA35* is involved in the regulation of apoptosis and proliferation of smooth muscle cells, but a role in cancer has not been reported so far. *AKNA* was weakly underexpressed in oligodendrogliomas with 9q deletion in comparison to normal brain tissue. *AKNA* encodes an AT-hook transcription factor that is known to specifically activate the expression of the *CD40* receptor and its ligand *CD40L/CD154*. *AKNA* has been suggested to play a role in inflammation and neoplastic transformations [18]. A polymorphism within the *AKNA* gene has been associated with an increased risk of cervical cancer [19]. *FBXW2* was underexpressed in oligodendrogliomas with 9q deletion compared to normal brain tissue. *FBXW2* encodes a F-box protein involved in ubiquitin-mediated degradation of proteins. *FBXW2* has recently been reported as tumor suppressor in lung cancer [20]. *EGFL7* was underexpressed in oligodendrogliomas with 9q deletion compared to normal brain tissue. *EGFL7* encodes a secreted endothelial cell protein with two epidermal growth factor-like domains. *EGFL7* expression has been found to be positively correlated with cell proliferation, aggressiveness, and angiogenesis in gliomas [21,22].

3.4 Chromosome 18: q-arm deletion in 20 of 133 oligodendrogliomas

CXXC1 was weakly underexpressed in oligodendrogliomas with 18q deletion compared to normal brain tissue. *CXXC1* encodes a transcriptional activator that binds specifically to non-methylated CpG motifs through its CXXC domain. No direct role of *CXXC1* in cancer has been reported so far.

3.5 Chromosome 7: p-arm duplication in 8 of 133 oligodendrogliomas

EIF3B was overexpressed in oligodendrogliomas with 7p duplication compared to normal brain tissue. *EIF3B* encodes a subunit of the eukaryotic translation initiation factor. Overexpression of *EIF3B* has been associated with malignant transformation of cells [23] and shown to be required for growth of bladder cancer [24]. A knockdown of *EIF3B* in a glioblastoma cell line has been shown to inhibit cell proliferation and to increase apoptosis [25].

3.6 Chromosome 7: q-arm duplication in 12 of 133 oligodendrogliomas

DMTF1 was overexpressed in oligodendrogliomas with 7q duplication in comparison to normal brain tissue. *DMTF1* encodes a transcription factor with a cyclin D-binding domain. *DMTF1* has been reported to act as tumor suppressor in bladder cancer inhibiting cell growth and cell cycle progression in dependency of miRNA-155 [26]. *CALD1* was overexpressed in oligodendrogliomas with 7q duplication compared to normal brain tissue. *CALD1* encodes a cytoskeleton-associated protein. Expression of different splice variants of *CALD1* have been reported to be involved in glioma neovascularization [27]. Overexpression of *CALD1* has been associated with lymph node metastasis and poor prognosis of squamous cell carcinoma patients [28]. *CALD1* has been associated with resistance to tamoxifen in estrogen receptor

positive recurrent breast cancer [29]. *DNAJB6* was overexpressed in oligodendrogliomas with 7q duplication in comparison to normal brain tissue. *DNAJB6* encodes a chaperone involved in protein folding and protein complex assembly. Reduced expression levels of *DNAJB6* have been found in aggressive breast cancer influencing tumor growth and metastasis [30]. *DNAJB6* is involved in tumor progression and metastasis formation [31]. Overexpression of *DNAJB6* has been reported to promote invasion of colorectal cancer [32]. Nuclear localization of *DNAJB6* has been associated with longer survival and reduced cell proliferation in esophageal cancer [33].

3.7 Chromosome 11: q-arm duplication in 6 of 133 oligodendrogliomas

FAU was overexpressed in oligodendrogliomas with 11q duplication compared to normal brain tissue. *FAU* encodes a fusion protein consisting of a ubiquitin-like protein and ribosomal protein. *FAU* has been reported as tumor suppressor in breast cancer regulating apoptosis [34]. *MAML2* was strongly overexpressed in oligodendrogliomas with 11q duplication compared to normal brain tissue. *MAML2* encodes a mastermind-like protein that can bind to the intracellular domain of notch receptors. Fusions of *MAML2* with other genes have been linked with tumor development in different types of cancers [35–37]. *NRGN* was highly underexpressed in oligodendrogliomas with 11q duplication compared to normal brain tissue. *NRGN* encodes a postsynaptic protein kinase substrate that binds calmodulin in the absence of calcium. Thus, it is likely that this gene may not play an important role for the development of oligodendrogliomas and may rather represent an artifact resulting from the comparison to normal brain tissue. Still, overexpression of *NRGN* has been suggested to have an oncogenic role in T-cell lymphomas [38].

4 Text S4: Impact of rare gene copy number mutations on metabolic pathways

In analogy to the characterization of genes with strong impact on the expression of cancer-relevant signaling pathway genes (Text S3), we characterized potential tumor gene candidates within chromosomal regions that were recurrently observed in smaller groups of oligodendrogliomas (see Tab. 1 of main manuscript; deletions: 4q, 9q, 13q, 15q, 18q; duplications: 7p, 7q, 11q). We first determined for each subset of oligodendrogliomas with a specific chromosomal arm mutation all differentially expressed genes in comparison to normal brain tissue (q-value ≤ 0.05, see main manuscript for general details to gene expression analysis). We next analyzed all differentially expressed genes of each mutated chromosomal arm with our network-based impact quantification strategy to identify differentially expressed genes within these regions that had a strong impact on the expression of metabolic pathway genes (Tab. S7, paired Wilcoxon rank sum tests, q-value ≤ 0.1, see main manuscript for general details to impact quantification). We were able to identify putative high-impact tumor candidate genes on 7p, 11q, 15q, and 18q. We again performed in-depth literature searches and analyzed corresponding gene annotations to identify potential cancer-relevant functions of these genes. In the following, we summarize our findings for individual high-impact candidate genes identified on the different mutated chromosomal arms.

4.1 Differentially expressed genes directly affected by rare chromosomal arm mutations with strong impact on metabolic pathways

Gene	Location	Gene expression	Impact in 10^{-3}
COX5A	15q (loss)	-1.84	1.8
PKM2	15q (loss)	-1.30	1.3
ATP5A1	18q (loss)	-0.86	1.0
MBP	18q (loss)	-5.05	0.8
GARS	7p (gain)	-0.14	0.5
PYGM	11q (gain)	-2.39	1.0
UBXN1	11q (gain)	1.35	0.4
FAU	11q (gain)	1.51	0.8
ACAT1	11q (gain)	1.25	1.3
SDHD	11q (gain)	0.72	0.7
TMEM25	11q (gain)	-0.84	0.5
NRGN	11q (gain)	-5.28	1.1

Predicted high-impact genes located on chromosomal arms rarely mutated in some oligodendrogliomas (Tab. S7, q-value ≤ 0.1). Gene expression: average $\log_2$-ratio of tumor to normal. loss: deletion of chromosomal arm. gain: duplication of chromosomal arm.

4.2 Chromosome 15: q-arm deletion in 12 of 133 oligodendrogliomas

COX5A was underexpressed in oligodendrogliomas with 15q deletion compared to normal brain tissue. *COX5A* encodes for the cytochrome C oxidase subunit 5A involved in the transfer of electrons from cytochrome c to molecular oxygen. A knockdown of *COX5A* has been reported to suppress migration and invasion of lung cancer cells and increased *COX5A* expression has been found to be associated with poor prognosis of lung adenocarcinomas [39]. *PKM2* was underexpressed in oligodendrogliomas with 15q deletion compared to normal brain tissue. *PKM2* encodes a pyruvate kinase that catalyzes the transfer of a phosphoryl group from phosphoenolpyruvate to ADP, generating ATP and pyruvate. *PKM2* is involved in glycolysis and has a general role in caspase-independent cell death of tumor cells. *PKM2* has been found to control glioma cell death and differentiation [40]. *PKM2* has been reported to promote glucose metabolism and cell growth of gliomas [41]. Known roles of *PKM2* in cancer were reviewed in [42].

4.3 Chromosome 18: q-arm deletion in 20 of 133 oligodendrogliomas

ATP5A1 was underexpressed in oligodendrogliomas with 18q deletion compared to normal brain tissue. *ATP5A1* encodes a subunit of the mitochondrial ATP synthase. *ATP5A1* has been found to be highly expressed in glioblastoma cells and in endothelial cells of the microvasculature [43]. *ATP5A1* has been reported to be downregulated in renal carcinoma [44]. *MBP* was strongly underexpressed in oligodendrogliomas with 18q deletion compared to normal brain tissue. *MBP* encodes a major constituent of the myelin sheath of oligodendrocytes and Schwann cells in the nervous system. *MBP* expression has been reported for oligodendrogliomas more than two decades ago [45]. Our finding indicate that the loss of *MBP* expression in a subset of oligodendrogliomas has downstream impacts on the expression of metabolic pathway genes.

4.4 Chromosome 7: p-arm duplication in 8 of 133 oligodendrogliomas

GARS was weakly underexpressed in oligodendrogliomas with 7p duplication compared to normal brain tissue. *GARS* encodes a glycyl-tRNA synthetase that charges tRNAs with their corresponding amino

acids. A clear role of *GARS* in cancer is not known so far, but cancer-associated activities of aminoacyl-tRNA synthetases have been reported for glioblastomas including *GARS* [46].

4.5 Chromosome 11: q-arm duplication in 6 of 133 oligodendrogliomas

PYGM was underexpressed in oligodendrogliomas with 11q duplication in comparison to normal brain tissue. *PYGM* encodes a phosphorylase involved in glycogenolysis. Mutations of *PYGM* have been reported for breast cancer and reduced expression was associated with poor relapse-free survival [47]. *UBXN1* was overexpressed in oligodendrogliomas with 11q duplication in comparison to normal brain tissue. *UBXN1* encodes a ubiquitin-binding protein. *UBXN1* has been found to be a negative regulator of NF-kB signaling [48]. *UBXN1* has been found to inhibit the tumor suppressor *BRAC1* [49]. Tumor suppressor and oncogenic functions of *UBXN1* have been reported [50]. *FAU* was overexpressed in oligodendrogliomas with 11q duplication compared to normal brain tissue. *FAU* encodes a fusion protein consisting of a ubiquitin-like protein and a ribosomal protein. *FAU* has been reported as tumor suppressor in breast cancer regulating apoptosis [34]. We previously also predicted *FAU* as high-impact gene that strongly influenced the expression of signaling pathways (Text S3). *ACAT1* was overexpressed in oligodendrogliomas with 11q duplication compared to normal brain tissue. *ACAT1* encodes a mitochondrially localized acetyl-CoA acetyltransferase. Inhibition of the enzymatic activity of *ACAT1* by Avasimibe inhibited cell growth by inducing cell cycle arrest and apoptosis in glioblastoma cell lines [51, 52]. Inhibition of *ACAT1* has recently been shown to suppress growth and metastasis of pancreatic cancer [53]. *SDHD* was overexpressed in oligodendrogliomas with 11q duplication compared to normal brain tissue. *SDHD* encodes the succinate dehydrogenase complex D, which is essential for the oxidation of succinate. Germline mutations of *SDHD* have been reported for head and neck paragangliomas [54]. Generally, the succinate dehydrogenase consists of four subunits (*SDHA*, *SDHB*, *SDHC*, *SDHD*), has a general role in cellular energy metabolism, and is known to act as tumor suppressor [11]. We already found the high-impact gene *SDHB* located on the p-arm of chromosome 1 to be underexpressed in oligodendrogliomas compared to normal brain (see Fig. 4b main manuscript). Thus, it is likely that the succinate dehydrogenase does not function properly in oligodendrogliomas. Succinate dehydrogenase deficiency induces pseudohypoxia and leads to the accumulation of succinate inhibiting alpha-ketoglutarate-dependent enzymes [11]. This may further support the epigenetic reprogramming triggered by the *IDH*-mutation present in each oligodendroglioma [12, 13]. A potential molecular function of overexpressed *SDHD* in a subset of oligodendrogliomas is not known so far. The overexpression of *SDHD* might represent a response of oligodendroglioma cells with 11q duplication to the underexpression of *SDHB*. Interestingly, activation of the expression of the tumor suppressor *CDKN1A* in response to the loss of *SDHD* expression has been reported [55]. Thus, overexpressed *SDHD* might counteract the expression of *CDKN1A* to support cell proliferation. *TMEM25* was underexpressed in oligodendrogliomas with 11q duplication compared to normal brain tissue. *TMEM25* encodes a trans-trans-membrane protein. Expression of *TMEM25* has been found to be correlated with better prognosis of breast cancer patients [56]. Epigentic downregulation of *TMEM25* has been reported for colorectal cancer [57]. *NRGN* was highly underexpressed in oligodendrogliomas with 11q duplication compared to normal brain tissue. *NRGN* encodes a postsynaptic protein kinase substrate that binds calmodulin in the absence of calcium. We previously predicted *NRGN* as high-impact gene that strongly influenced the expression of signaling pathways and discussed this gene in Text S3.

5 Supporting figures

5.1 Figure S1: Hierarchical clustering of histologically classified oligodendrogliomas based on gene copy number data

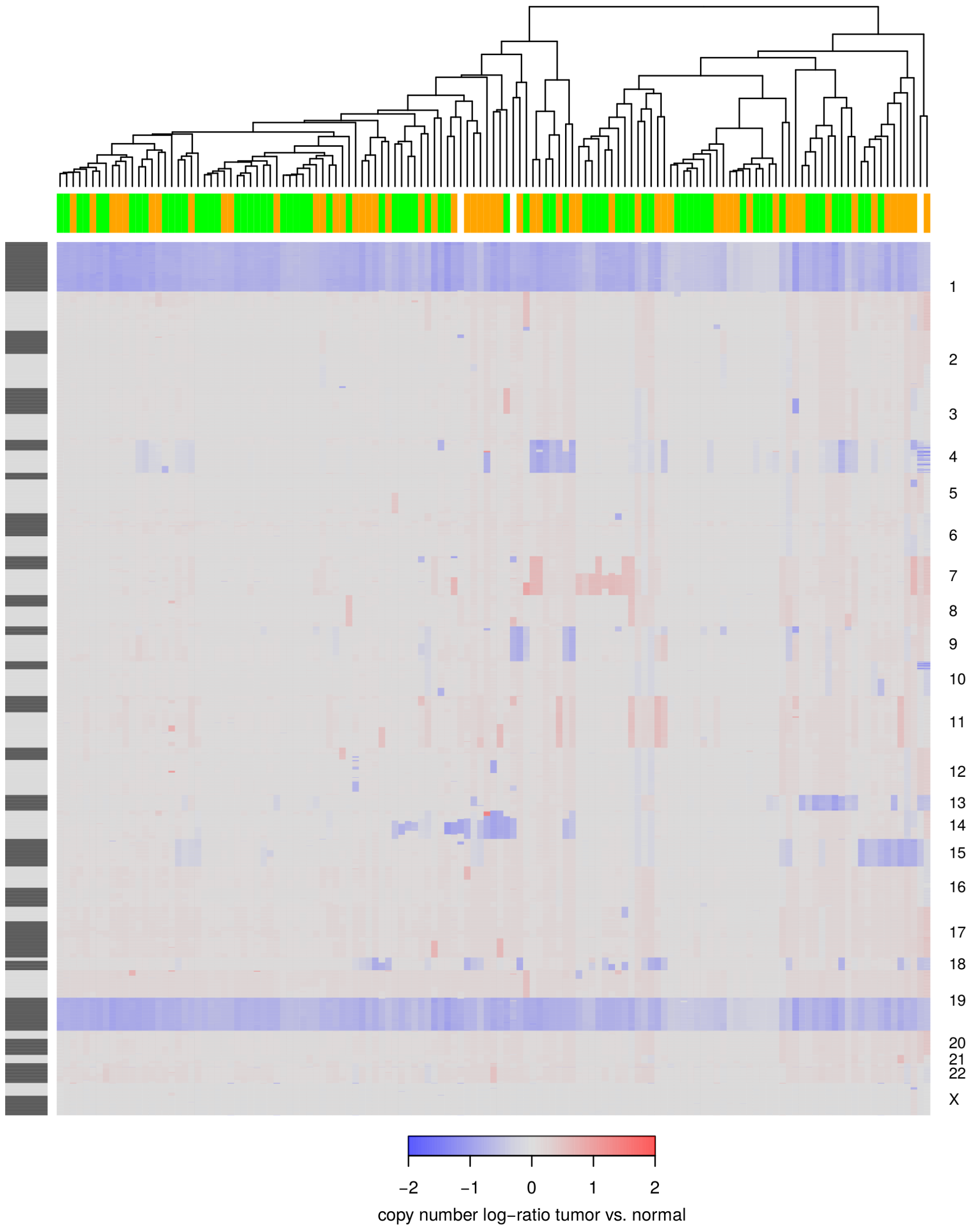

Figure S1: Heatmap of genome-wide gene copy number log-ratios of 133 oligodendrogliomas with 1p/19q-codeletion (columns) compared to normal DNA for 12,285 genes (rows); blue: deletions, gray: unchanged, red: duplications. Tumors of WHO grade II are labeled in green, WHO grade III is labeled in orange, and white is used if no WHO grade was assigned. Chromosome names are shown to the right, and chromosomal arms are highlighted by alternating dark grey and grey bars to the left. The characteristic oligodendroglioma-specific 1p/19q co-deletion and other less frequent chromosomal mutations are clearly visible.

5.2 Figure S2: Survival analysis of oligodendrogliomas with 1p/19q co-deletion

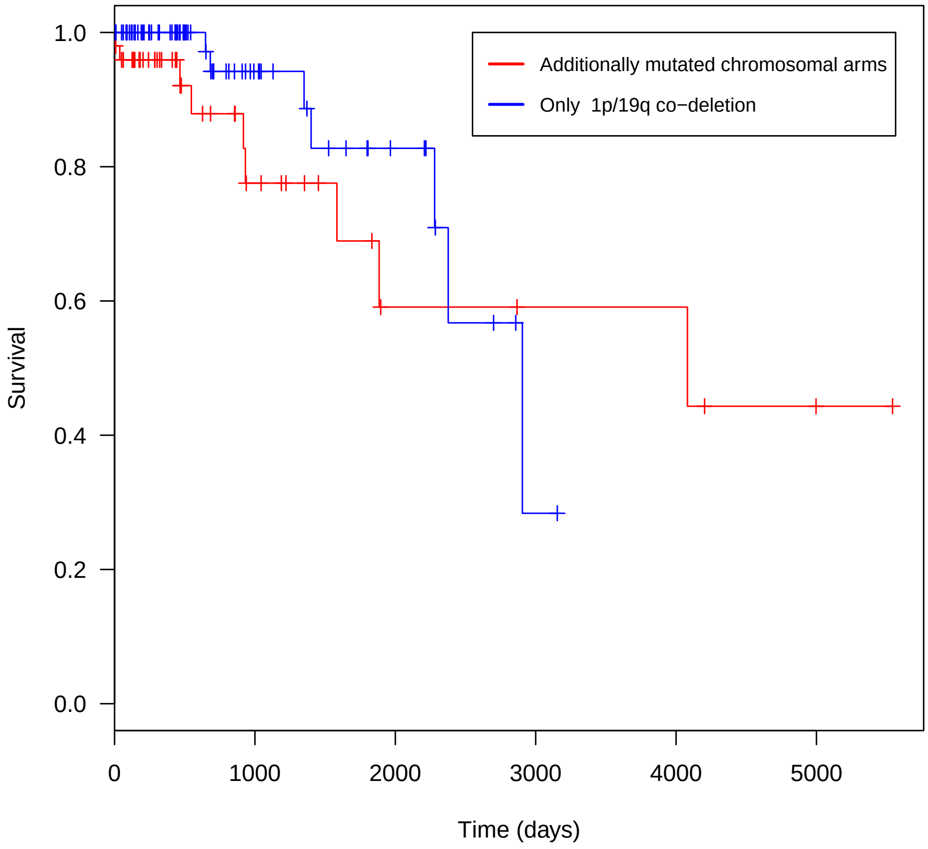

Figure S2: Kaplan-Meier plots comparing patient survival for oligodendrogliomas that only show a 1p/19q co-deletion to oligodendrogliomas with 1p/19q co-deletion (blue curve) and additionally mutated chromosomal arms (red curve; deletions: 4q, 9q, 13q, 15q, or 18q; duplications: 7p, 7q, 11q). See Fig. S1 for gene copy number profiles of considered patients. No significant difference in survival between both patient groups were observed (log-rank test: $P = 0.37$, 55 patients with additional chromosomal arm mutations vs. 78 patients with 1p/19q co-deletion only). Survival data were taken from Table S1 of [58].

5.3 Figure S3: Learned gene regulatory networks

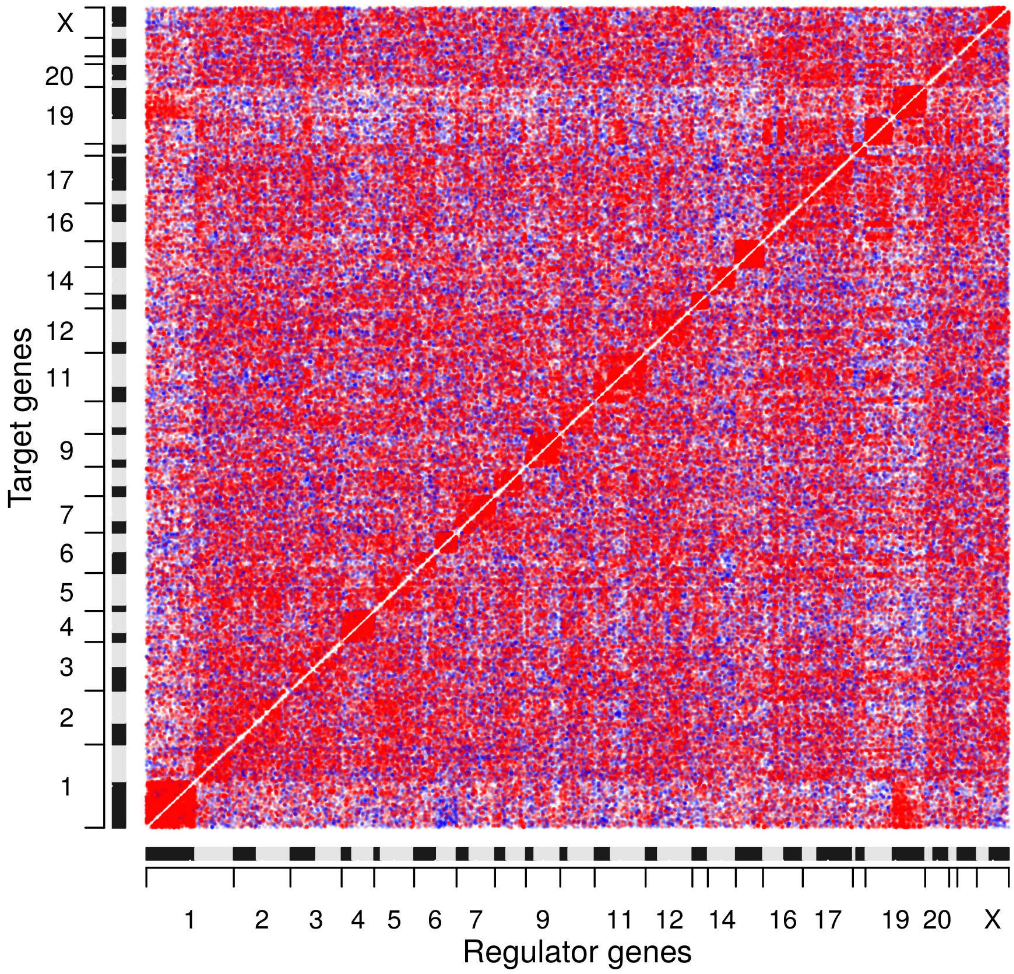

Figure S3: Integrative visualization of the ten networks derived from TCGA data. Included are the utilized significant links (q-value $\leq$ 0.01) of each network cleaned up for local regulators 50 genes up- and downstream of each target gene. Links from regulator to target genes are visualized by colored dots (red: activator, blue: inhibitor). Frequently observed links among the ten networks have darker dot colors than less frequently observed links. Regulator and target genes are aligned in chromosomal order from 1 to X and corresponding chromosomal arms are displayed by alternating black and grey bars. The red band around the main diagonal represents local chromosomal activator links. There are more links between genes of the same chromosomal arm than between genes of different chromosomal arms (Fisher's exact test, $P < 1 \times 10^{-100}$). A similar band has also previously been observed in genome-wide gene regulatory networks learned from human gene expression profiles [59] or learned from human cancer cell lines [60]. A specific characteristic of the networks is the enrichment of activator links between the chromosomal arms 1p and 19q (Fisher's exact test, $P < 1 \times 10^{-50}$).

5.4 Figure S4: Genes with many outgoing links in learned networks

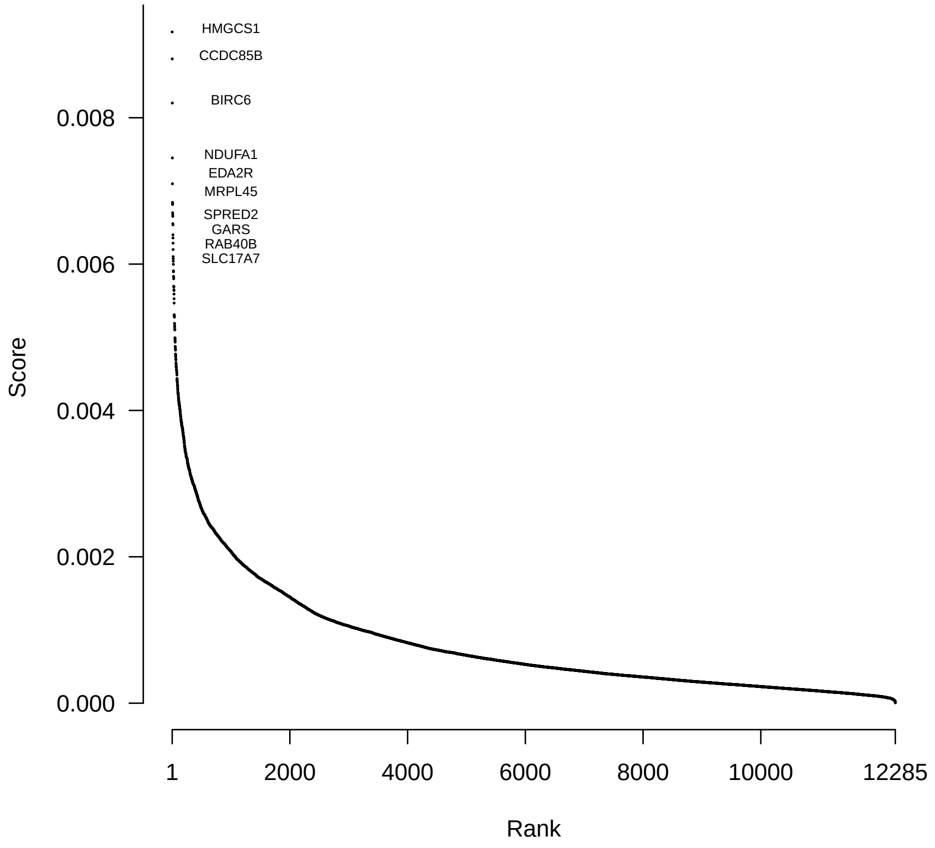

Figure S4: Ranking of genes across the ten learned networks taking the stability of links across the networks and their number of outgoing links to other genes into account. Genes with higher scores have a greater number of more frequently predicted outgoing links to genes across the ten networks than genes with lower scores (see main manuscript for details). Names of the ten genes with highest scores are shown.

5.5 Figure S5: Gene expression prediction quality of individual networks

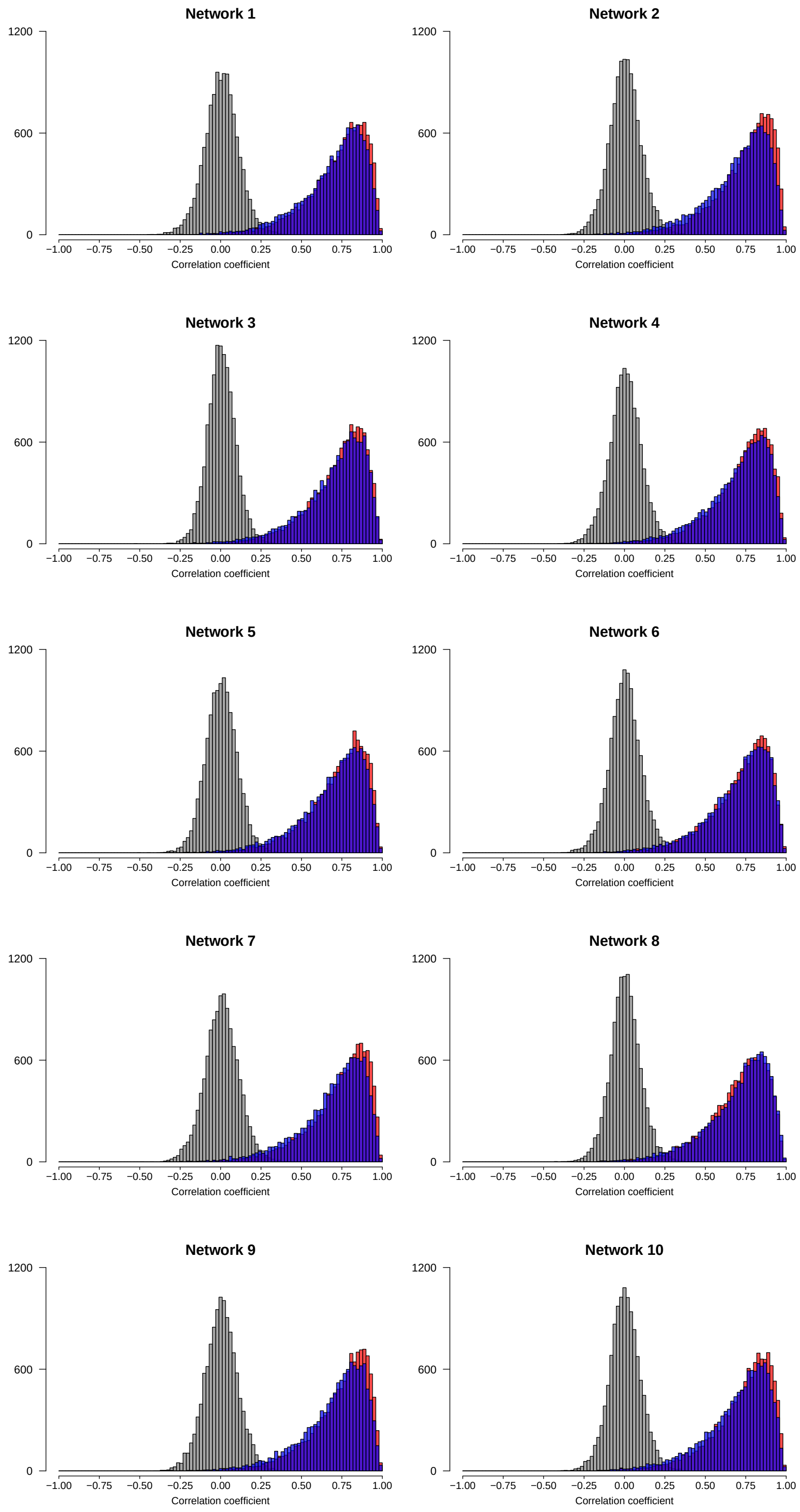

Figure S5: Prediction of gene expression levels by oligodendroglioma-specific gene regulatory networks. Each network was analyzed for its performance to predict the expression levels of the 12,285 of independent oligodendroglioma (red) and oligoastrocytoma (blue) test samples. Corresponding histograms show obtained gene-specific correlations between predicted and measured gene expression levels. Both histograms (red, blue) are always clearly shifted into the positive range. Median correlations of 25 random networks of same complexity as the corresponding learned oligodendroglioma-specific network are shown in grey. The prediction quality of individual networks was significantly better than under random networks ($P < 2.2 \times 10^{-16}$ for each network, Wilcoxon rank sum test).

5.6 Figure S6: Downstream effects of high-impact genes on individual cancer-relevant signaling and metabolic pathways

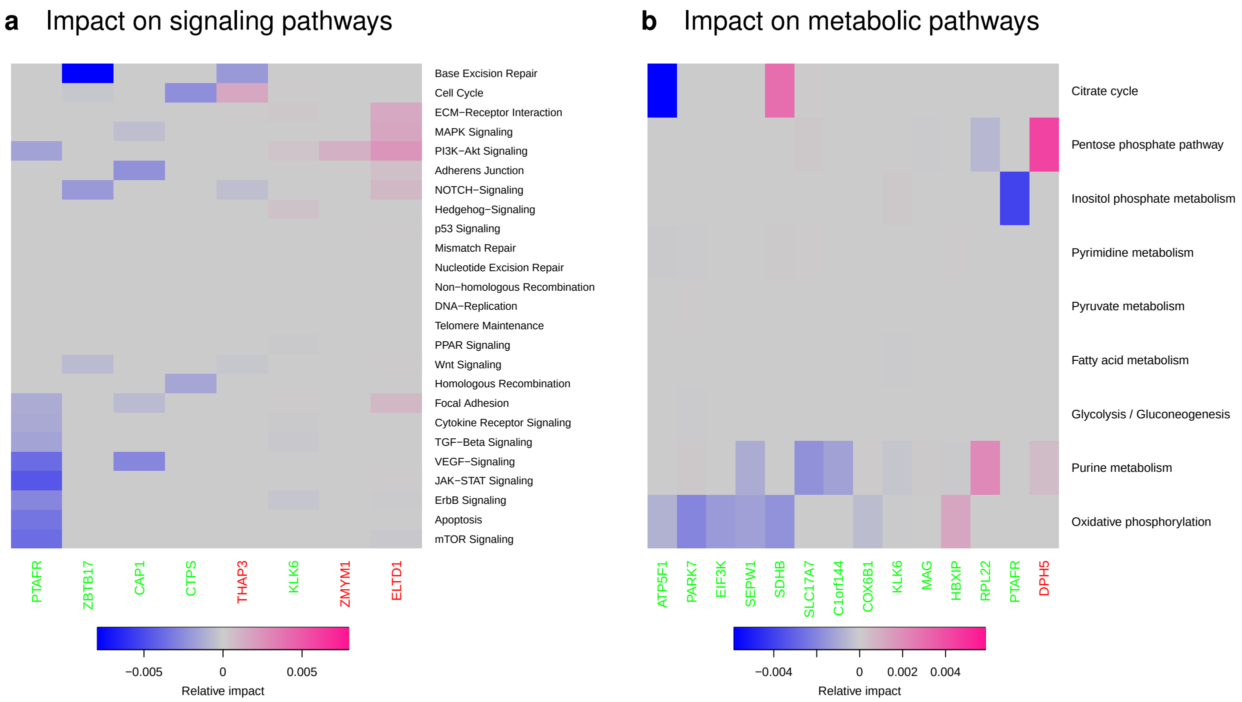

Figure S6: Potential regulatory downstream effects of high-impact genes of the 1p/19q region on individual signaling and metabolic pathways. Predicted regulatory impacts of oligodendroglioma-specific candidate genes of the 1p/19q co-deletion region (main manuscript: Fig. 4) on specific specific known cancer-relevant signaling (a) and metabolic (b) pathways. Underexpressed candidate genes whose average expression level was lower in oligodendrogliomas compared to normal brain are colored in green and overexpressed candidate genes are colored in red. Corresponding putative regulatory impacts of candidate genes on specific pathways are specified by relative impacts highlighted in blue (negative impact) if a gene under consideration of its underlying expression state (under- or overexpressed) tends to inhibit the expression of genes of a specific pathway and highlighted in pink (positive impact) if the gene under consideration of its expression state tends to activate the expression of genes of a specific pathway.

5.7 Figure S7: Impacts of FUBP1 and CIC underexpression on cancer-relevant signaling and metabolic pathways

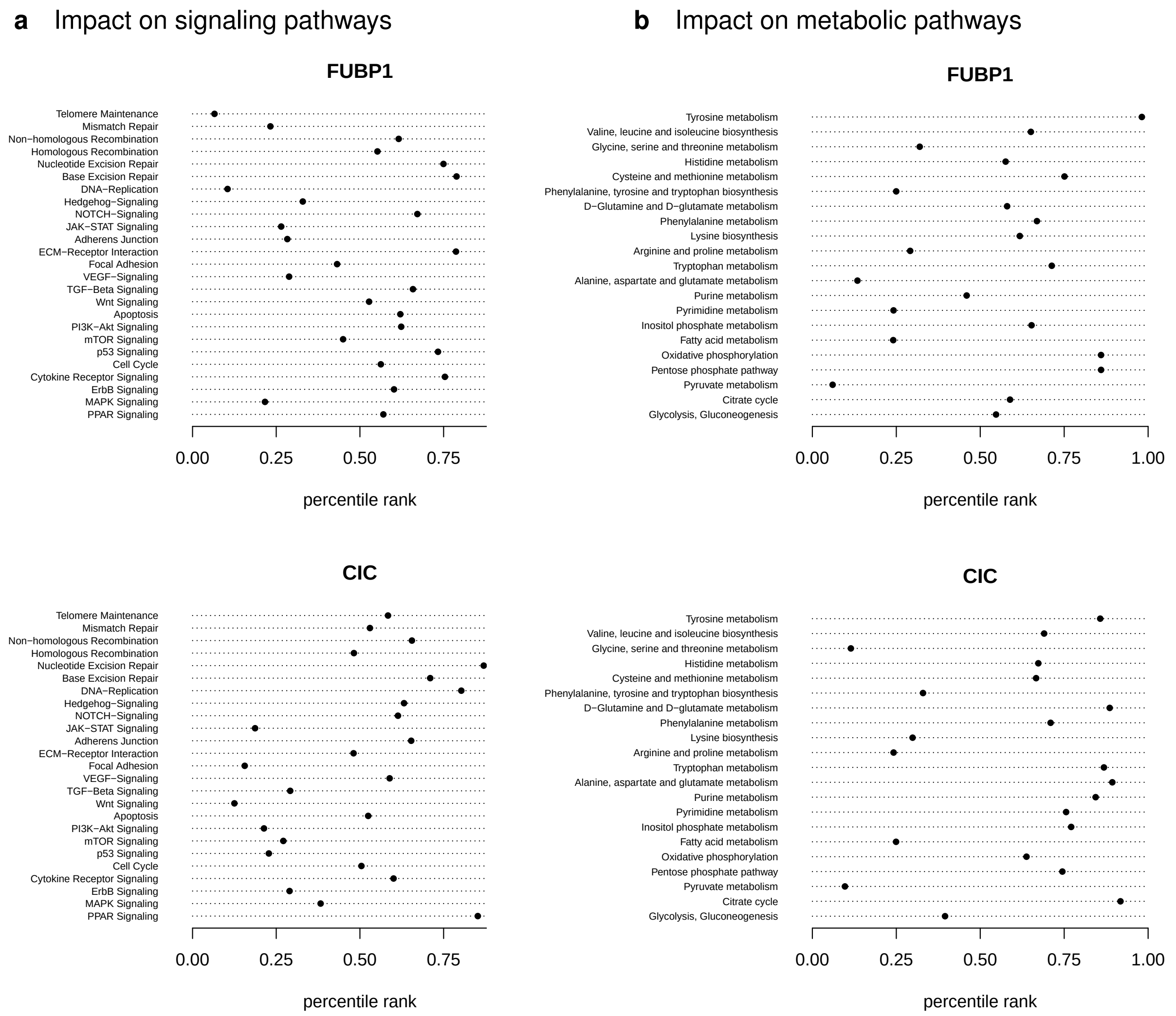

Figure S7: Potential impacts of *FUBP1* and *CIC* underexpression on cancer-relevant signaling (a) and metabolic pathways (b). Predicted regulatory impacts of underexpressed *FUBP1* and *CIC* were ranked according to the corresponding impacts of all other 12,284 genes. Ranking of *FUBP1* or *CIC* relative to the other genes is quantified by percentile ranks representing the proportion of genes with impact values equal or less than *FUBP1* or *CIC*. Note that the impacts behind the ranking range from strongly inhibitory (percentile rank 0) to strongly activating (percentile rank 1). Rankings of *FUBP1* and *CIC* for individual pathways are highlighted by black dots.

References

[1] M. Seifert and A. Beyer. regNet: An R package for network-based propagation of gene expression alterations. *Bioinformatics*, 34(2):308–311, 2018.

[2] R. A. Towner, R. L. Jensen, H. Colman, B. Vaillant, N. Smith, R. Casteel, et al. ELTD1, a potential new biomarker for gliomas. *Neurosurgery*, 72(1):77–91, 2013.

[3] M. Masiero, F. C. Simoes, H. D. Han, C. Snell, T. Peterkin, E. Bridges, et al. A core human primary tumor angiogenesis signature identifies the endothelial orphan receptor ELTD1 as a key regulator of angiogenesis. *Cancer Cell*, 24(2):229–241, 2013.

[4] J. Ziegler, R. Pody, P. Coutinho de Souza, B. Evans, D. Saunders, N. Smith, et al. ELTD1, an effective anti-angiogenic target for gliomas: preclinical assessment in mouse GL261 and human G55 xenograft glioma models. *Neuro-Oncology*, 19(2):175–185, 2017.

[5] M. Safran, I. Dalah, J. Alexander, N. Rosen, T. Iny Stein, M. Shmoish, et al. GeneCards version 3: the human gene integrator. *Database*, 2010:baq020, 2010.

[6] J. Chen, T. Lan, W. Zhang, L. Dong, N. Kang, S. Zhang, et al. Platelet-activating factor receptor-mediated PI3k/AKT activation contributes to the malignant development of esophageal squamous cell carcinoma. *Oncogene*, 34(40):5114–5127, 2015.

[7] J. M. Cuezva, M. Krajewska, M. L. de Heredia, S. Krajewski, G. Santamaria, H. Kim, et al. The bioenergetic signature of cancer: a marker of tumor progression. *Cancer Res*, 62(22):6674–81, 2002.

[8] O. Warburg. On the origin of cancer cells. *Science*, 123(3191):309–14, 1956.

[9] I. M. Willers and J. M. Cuezva. Post-transcriptional regulation of the mitochondrial H(+)-ATP synthase: a key regulator of the metabolic phenotype in cancer. *Biochim Biophys Acta*, 1807(6):543–51, 2011.

[10] S. Richter, M. Peitzsch, E. Rapizzi, J. W. Lenders, N. Qin, et al. Krebs cycle metabolite profiling for identification and stratification of pheochromocytomas/paragangliomas due to succinate dehydrogenase deficiency. *J Clin Endocrinol Metab*, 99(10):3903–11, 2014.

[11] C. Bardella, P. J. Pollard, and I. Tomlinson. SDH mutations in cancer. *Biochim Biophys Acta*, 1807(11):1432–43, 2011.

[12] D. N. Louis, A. Perry, G. Reifenberger, A. von Deimling, D. Figarella-Branger, W. K. Cavenee, et al. The 2016 World Health Organization Classification of Tumors of the Central Nervous System: a summary. *Acta Neuropathologica*, 131(6):803–820, 2016.

[13] A. Cohen, S. Holmen, and H. Colman. IDH1 and IDH2 mutations in gliomas. *Curr Neurol Neurosci Rep*, 13(5):345, 2013.

[14] A. Kamoun, A. Idbaih, C. Dehais, N. Elarouci, C. Carpentier, E. Letouzé, et al. Integrated multi-omics analysis of oligodendroglial tumours identifies three subgroups of 1p/19q co-deleted gliomas. *Nature Communications*, 7:11263, 2016.

[15] I. Tirosh, A. S. Venteicher, C. Hebert, L. E. Escalante, A. P. Patel, K. Yizhak, et al. Single-cell RNA-seq supports a developmental hierarchy in human oligodendroglioma. *Nature*, 539(539):309–313, 2016.

[16] M. Kinoshita, T. Nakamura, M. Ihara, T. Haraguchi, Y. Hiraoka, K. Tashiro, et al. Identification of human endomucin-1 and -2 as membrane-bound O-sialoglycoproteins with anti-adhesive activity. *FEBS Lett*, 499(1-2):121–126, 2001.

[17] G. Hirano, H. Izumi, Y. Yasuniwa, S. Shimajiri, W. Ke-Yong, Y. Sasagiri, et al. Involvement of riboflavin kinase expression in cellular sensitivity against cisplatin. *Int J Oncol*, 38(4):893–902, 2011.

[18] A. R. Moliterno and L. M. S. Resar. AKNA: Another AT-hook transcription factor "hooking-up" with inflammation. *Cell Res*, 21(11):1528–30, 2011.

[19] G. Perales, A. I. Burguete-Garcia, J. Dimas, M. Bahena-Roman, V. H. Bermudez-Morales, J. Moreno, et al. A polymorphism in the AT-hook motif of the transcriptional regulator AKNA is a risk factor for cervical cancer. *Biomarkers*, 15(5):470–4, 2010.

[20] J. Xu, W. Zhou, F. Yang, G. Chen, H. Li, Y. Zhao, et al. The β-TrCP-FBXW2-SKP2 axis regulates lung cancer cell growth with FBXW2 acting as a tumour suppressor. *Nat Commun*, 8(8):14002, 2017.

[21] C. Huang, X. Yuan, Y. Wan, F. Liu, X. Chen, X. Zhan, et al. VE-statin/Egfl7 expression in malignant glioma and its relevant molecular network. *Int J Clin Exp Pathol*, 7(3):1022–1031, 2014.

[22] C.-S. Wang, F.-Y.-F.and Kang, S.-Y. Wang-Gou, C.-H. Huang, C.-Y. Feng, and X.-J. Li. EGFL7 is an intercellular EGFR signal messenger that plays an oncogenic role in glioma. *Cancer Lett*, 384:9–18, 2017.

[23] J. W. Hershey. The role of eIF3 and its individual subunits in cancer. *Biochim Biophys Acta*, 1849(7):792–800, 2015.

[24] H. Wang, Y. Ru, M. Sanchez-Carbayo, X. Wang, J. S. Kieft, and D. Theodorescu. Translation initiation factor eIF3b expression in human cancer and its role in tumor growth and lung colonization. *Clin Cancer Res*, 19(11):2850–60, 2013.

[25] H. Liang, X. Ding, C. Zhou, Y. Zhang, M. Xu, C. Zhang, et al. Knockdown of eukaryotic translation initiation factors 3B (EIF3B) inhibits proliferation and promotes apoptosis in glioblastoma cells. *Neurol Sci*, 33(5):1057–62, 2012.

[26] Y. Peng, W. Dong, T. X. Lin, G. Z. Zhong, B. Liao, and B. Wang. MicroRNA-155 promotes bladder cancer growth by repressing the tumor suppressor DMTF1. *Oncotarget*, 6(18):16043–58, 2015.

[27] P. P. Zheng, A. M. Sieuwerts, T. M. Luider, M. van der Weiden, P. A. Sillevis-Smitt, and J. M. Kros. Differential expression of splicing variants of the human caldesmon gene (CALD1) in glioma neovascularization versus normal brain microvasculature. *Am J Pathol*, 164(6):2217–28, 2004.

[28] K. P. Chang, C. L. Wang, H. K. Kao, Y. Liang, S. C. Liu, L. L. Huang, et al. Overexpression of caldesmon is associated with lymph node metastasis and poorer prognosis in patients with oral cavity squamous cell carcinoma. *Cancer*, 119(22):4003–11, 2013.

[29] T. De Marchi, A. M. Timmermans, M. Smid, M. P. Look, C. Stingl, and M. Opdam. Annexin-A1 and caldesmon are associated with resistance to tamoxifen in estrogen receptor positive recurrent breast cancer. *Oncotarget*, 7(3):3098–110, 2016.

[30] A. Mitra, R. A. Fillmore, B. J. Metge, M. Rajesh, Y. Xi, J. King, et al. Large isoform of MRJ (DNAJB6) reduces malignant activity of breast cancer. *Breast Cancer Res*, 10(2):R22, 2008.

[31] E. Meng, L. A. Shevde, and R. S. Samant. Emerging roles and underlying molecular mechanisms of DNAJB6 in cancer. *Oncotarget*, 7(33):53984–53996, 2016.

[32] T. T. Zhang, Y. Y. Jiang, L. Shang, Z. Z. Shi, J. W. Liang, and Z. Wang. Overexpression of DNAJB6 promotes colorectal cancer cell invasion through an IQGAP1/ERK-dependent signaling pathway. *Mol. Carcinog.*, 54:1205–13, 2015.

[33] V. Z. Yu, V. C. Wong, W. Dai, J. M. Ko, A. K. Lam, K. W. Chan, et al. Nuclear localization of DNAJB6 is associated with survival of patients with esophageal cancer and reduces AKT signaling and proliferation of cancer cells. *Gastroenterology*, 149(7):1825–1836, 2015.

[34] M. R. Pickard, A. R. Green, I. O. Ellis, C. Caldas, V. L. Hedge, and M. Mourtada-Maarabouni. Dysregulated expression of Fau and MELK is associated with poor prognosis in breast cancer. *Breast Cancer Res.*, 11(4):R60, 2009.

[35] T. Komiya, Y. Park, S. Modi, A.B. Coxon, H. Oh, and F. J. Kaye. Sustained expression of Mect1-Maml2 is essential for tumor cell growth in salivary gland cancers carrying the t(11;19) translocation. *Oncogene*, 25(45):6128–32, 2006.

[36] D. Bell, M. A. Luna, R. S. Weber, F. J. Kaye, and A. K. El-Naggar. CRTC1/MAML2 fusion transcript in Warthin's tumor and mucoepidermoid carcinoma: evidence for a common genetic association. *Genes Chromosomes Cancer*, 47(4):309–14, 2008.

[37] T. Nakayama, S. Miyabe, M. Okabe, H. Sakuma, K. Ijichi, and Y. Hasegawa. Clinicopathological significance of the CRTC3-MAML2 fusion transcript in mucoepidermoid carcinoma. *Mod Pathol.*, 22(12):1575–81, 2009.

[38] A. A. Nielsen, K. R. Kjartansdottir, M. H. Rasmussen, A. B. Sorensen, B. Wang, M. Wabl, et al. Activation of the brain-specific neurogranin gene in murine T-cell lymphomas by proviral insertional mutagenesis. *Gene*, 442(1-2):55–62, 2009.

[39] W. L. Chen, K. T. Kuo, T. Y. Chou, C. L. Chen, C. H. Wang, Y. H. Wei, et al. The role of cytochrome c oxidase subunit Va in non-small cell lung carcinoma cells: association with migration, invasion and prediction of distant metastasis. *BMC Cancer*, 12:273, 2012.

[40] M. Morfouace, L. Lalier, L. Oliver, M. Cheray, C. Pecqueur, P. F. Cartron, et al. Control of glioma cell death and differentiation by PKM2-Oct4 interaction. *Cell Death Dis*, 5:e1036, 2014.

[41] W. Luan, Y. Wang, X. Chen, Y. Shi, J. Wang, J. Zhang, et al. PKM2 promotes glucose metabolism and cell growth in gliomas through a mechanism involving a let-7a/c-Myc/hnRNPA1 feedback loop. *Oncotarget*, 6(15):13006–18, 2015.

[42] G. Dong, Q. Mao, W. Xia, Y. Xu, J. Wang, L. Xu, et al. PKM2 and cancer: The function of PKM2 beyond glycolysis. *Oncol Lett*, 11(3):1980–86, 2016.

[43] G. Xu and J. Y. Li. ATP5A1 and ATP5B are highly expressed in glioblastoma tumor cells and endothelial cells of microvascular proliferation. *J Neurooncol*, 126(3):405–13, 2016.

[44] M. Brüggemann, A. Gromes, M. Poss, D. Schmidt, N. Klümper, and Y. Tolkach. Systematic analysis of the expression of the mitochondrial ATP synthase (Complex V) subunits in clear cell renal cell carcinoma. *Transl Oncol*, 10(4):661–668, 2017.

[45] J. G. Golfinos, S. A. Norman, S. W. Coons, R. A. Norman, C. Ballecer, and A. C. Scheck. Expression of the genes encoding myelin basic protein and proteolipid protein in human malignant gliomas. *Clin Cancer Res*, 3(5):799–804, 1997.

[46] Y. W. Kim, C. Kwon, J. L. Liu, S. H. Kim, and S. Kim. Cancer association study of aminoacyl-trna synthetase signaling network in glioblastoma. *PLoS One*, 7(8):e40960, 2012.

[47] M. V. Dieci, V. Smutná, V. Scott, G. Yin, R. Xu, and P. Vielh. Whole exome sequencing of rare aggressive breast cancer histologies. *Breast Cancer Res Treat*, 156(1):21–32, 2016.

[48] Y.-B. Wang, B. Tan, R. Mu, Y. Chang, M. Wu, H.-Q. Tu, et al. Ubiquitin-associated domain-containing ubiquitin regulatory X (UBX) protein UBXN1 is a negative regulator of nuclear factor κb (nf-κb) signaling. *J Biol Chem*, 290(16):10395–10405, 2015.

[49] F. Wu-Baer, T. Ludwig, and R. Baer. The UBXN1 protein associates with autoubiquitinated forms of the BRCA1 tumor suppressor and inhibits its enzymatic function. *Mol Cell Biol*, 30(11):2787–2798, 2010.

[50] K. Rezvani. UBXD proteins: a family of proteins with diverse functions in cancer. *Int J Mol Sci*, 17(10):1724, 2016.

[51] S. Bemlih, M.-D. Poirier, and A. E. Andaloussi. Acyl-coenzyme A: cholesterol acyltransferase inhibitor Avasimibe affect survival and proliferation of glioma tumor cell lines. *Cancer Biology & Therapy*, 9(12):1025–1032, 2010.

[52] T. Ohmoto, K. Nishitsuji, N. Yoshitani, M. Mizuguchi, Y. Yanagisawa, H. Saito, et al. K604, a specific acyl-CoA:cholesterol acyltransferase 1 inhibitor, suppresses proliferation of U251-MG glioblastoma cells. *Mol Med Rep*, 12(4):6037–42, 2015.

[53] J. Li, D. Gu, S. S. Lee, B. Song, S. Bandyopadhyay, S. Chen, et al. Abrogating cholesterol esterification suppresses growth and metastasis of pancreatic cancer. *Oncogene*, 35(50):6378–6388, 2016.

[54] B. E. Baysal, J. E. Willett-Brozick, E. C. Lawrence, C. M. Drovdlic, S. A. Savul, D.R. McLeod, et al. Prevalence of SDHB, SDHC, and SDHD germline mutations in clinic patients with head and neck paragangliomas. *J Med Genet*, 39(3):178–83, 2002.

[55] A. Millán-Uclés, B. Diaz-Castro, P. Garcia-Flores, A. Báez, J. A. Pérez-Simón, J. López-Barneo, et al. A conditional mouse mutant in the tumor suppressor SdhD gene unveils a link between p21(WAF1/Cip1) induction and mitochondrial dysfunction. *PLoS One*, 9(1):e85528, 2014.

[56] P. Doolan, M. Clynes, S. Kennedy, J. P. Mehta, S. Germano, C. Ehrhardt, et al. TMEM25, REPS2 and Meis 1: favourable prognostic and predictive biomarkers for breast cancer. *Tumour Biol*, 30(4):200–9, 2009.

[57] S. Hrasovec, N. Hauptman, D. Glavac, F. Jelenc, M. Ravnik-Glavac, et al. Tmem25 is a candidate biomarker methylated and down-regulated in colorectal cancer. *Dis Markers*, 34(2):93–104, 2013.

[58] M. Ceccarelli, F. P. Barthel, T. M. Malta, T. S. Sabedot, S. R. Salama, B. A. Murray, et al. Molecular profiling reveals biologically discrete subsets and pathways of progression in diffuse glioma. *Cell*, 164(3):550–563, 2016.

[59] V. Belcastro, V. Siciliano, F. Gregoretti, P. Mithbaokar, G. Dharmalingam, S. Berlingieri, et al. Transcriptional gene network inference from a massive dataset elucidates transcriptome organization and gene function. *Nucleic Acids Res*, 39(20):8677–8688, 2011.

[60] M. Seifert, B. Friedrich, and A. Beyer. Importance of rare gene copy number alterations for personalized tumor characterization and survival analysis. *Genome Biology*, 17:204, 2016.
